# The evolution and functional divergence of the histone H2B family in plants

**DOI:** 10.1371/journal.pgen.1008964

**Published:** 2020-07-27

**Authors:** Danhua Jiang, Michael Borg, Zdravko J. Lorković, Sean A. Montgomery, Akihisa Osakabe, Ramesh Yelagandula, Elin Axelsson, Frédéric Berger

**Affiliations:** 1 Gregor Mendel Institute, Austrian Academy of Sciences, Vienna BioCenter, Dr. Bohr-Gasse, Vienna, Austria; 2 State Key Laboratory of Plant Genomics, Institute of Genetics and Developmental Biology, The Innovative Academy of Seed Design, Chinese Academy of Sciences, Beijing, China; 3 University of Chinese Academy of Sciences, Beijing, China; Fred Hutchinson Cancer Research Center, UNITED STATES

## Abstract

Chromatin regulation of eukaryotic genomes depends on the formation of nucleosome complexes between histone proteins and DNA. Histone variants, which are diversified by sequence or expression pattern, can profoundly alter chromatin properties. While variants in histone H2A and H3 families are well characterized, the extent of diversification of histone H2B proteins is less understood. Here, we report a systematic analysis of the histone H2B family in plants, which have undergone substantial divergence during the evolution of each major group in the plant kingdom. By characterising *Arabidopsis* H2Bs, we substantiate this diversification and reveal potential functional specialization that parallels the phylogenetic structure of emergent clades in eudicots. In addition, we identify a new class of highly divergent H2B variants, H2B.S, that specifically accumulate during chromatin compaction of dry seed embryos in multiple species of flowering plants. Our findings thus identify unsuspected diverse properties among histone H2B proteins in plants that has manifested into potentially novel groups of histone variants.

## Author summary

In addition to well-studied variants from core histones families H2A and H3, we report that land plants diversified their H2B family, leading to specialized H2B variants with specific patterns of expression, genomic distributions and properties.

## Introduction

The basic subunit of chromatin is the nucleosome, which contains an octamer core of histones H2A, H2B, H3 and H4 wrapped around 147bp of DNA [1]. The tight control of nucleosomal organization is critical for chromatin processes like transcription, DNA replication, repair and recombination [2–4]. Individual paralogous genes of each histone family often encode related but functionally distinct proteins, which are referred to as “histone variants” when they acquire convergent properties during evolution [5, 6].

Histone variants often differ by cell cycle or developmental stage-specific expression patterns [6–8]. For example, replicative histone H3.1/H3.2 are primarily incorporated into replicated chromatin during S-phase, whereas histone H3.3 functions as a replacement histone throughout the cell cycle during processes like transcription [2, 9–15]. CenH3/CENP-A is highly divergent from other H3 variants and is incorporated specifically within centromeric regions [16]. Atypical histone H3 variants also exist that have specific substitutions within their N-terminal tail [17, 18]. For instance, the sperm-specific histone H3.10 resists K27 methylation and helps reprogram H3K27me3 during *Arabidopsis* spermatogenesis [19–21]. H3.15 lacks K27 and is induced during wound regeneration in *Arabidopsis* [22]. Sperm-specific H3 variants have also evolved convergently in mammals, such as histone H3.5 and H3.T, which alter nucleosome properties and participate in sperm maturation [23, 24].

Animals and plants share several common H2A variants, including canonical H2A, H2A.Z and H2A.X. H2A.Z is predominantly associated with transcription [8, 25], while H2A.X is essential for DNA repair [26]. Vertebrate genomes also encode macroH2A, which is essential for development and heterochromatin organization [8, 27, 28]. Similarly, histone H2A.W in land plants is involved in heterochromatin organization [29–31]. Additional histone H2As have also evolved in mammals, including H2A.Bbd that is strongly expressed in testis and to a lesser degree in the brain [32, 33], as well as other H2As restricted to primate testes [34].

Compared with H3 and H2A, only a handful of H4 and H2B variants have been characterized [35]. Notable examples are the testis-specific TH2B [36], the sperm expressed H2B.W and *sub*H2B [37, 38], and the neuron-specific H2BE in mice [39]. An expanded set of H2Bs have been also identified in *Arabidopsis* [5] but apart from the analysis of post-translational modifications [40, 41], the extent of their functional diversification has not yet been determined. An investigation into the evolutionary origin of plant histone H2Bs is thus lacking and it remains unclear whether histone H2Bs qualify as histone variants.

Here, we report a systematic characterization of plant H2Bs and reveal high sequence divergence and evolutionary constraints within each major lineage of the plant kingdom. We reveal how *Arabidopsis* H2B expression varies across development and reveal a subset of H2Bs that are specifically expressed in reproductive tissues. Moreover, we identify a clade of highly divergent H2Bs in flowering plants that we propose as a new class of seed specific H2B.S variants. By characterizing H2Bs expressed in *Arabidopsis* somatic tissues, we identify a putative replacement histone H2B and reveal two groups with preferential deposition in heterochromatic and euchromatic regions of the genome. This report thus expands our knowledge of the evolutionary history and distinct properties of plant histone H2Bs, paving the way for mechanistic studies into their impact on chromatin structure and function.

## Results

### H2B sequences are highly divergent in plant phyla

**Fig 1:**
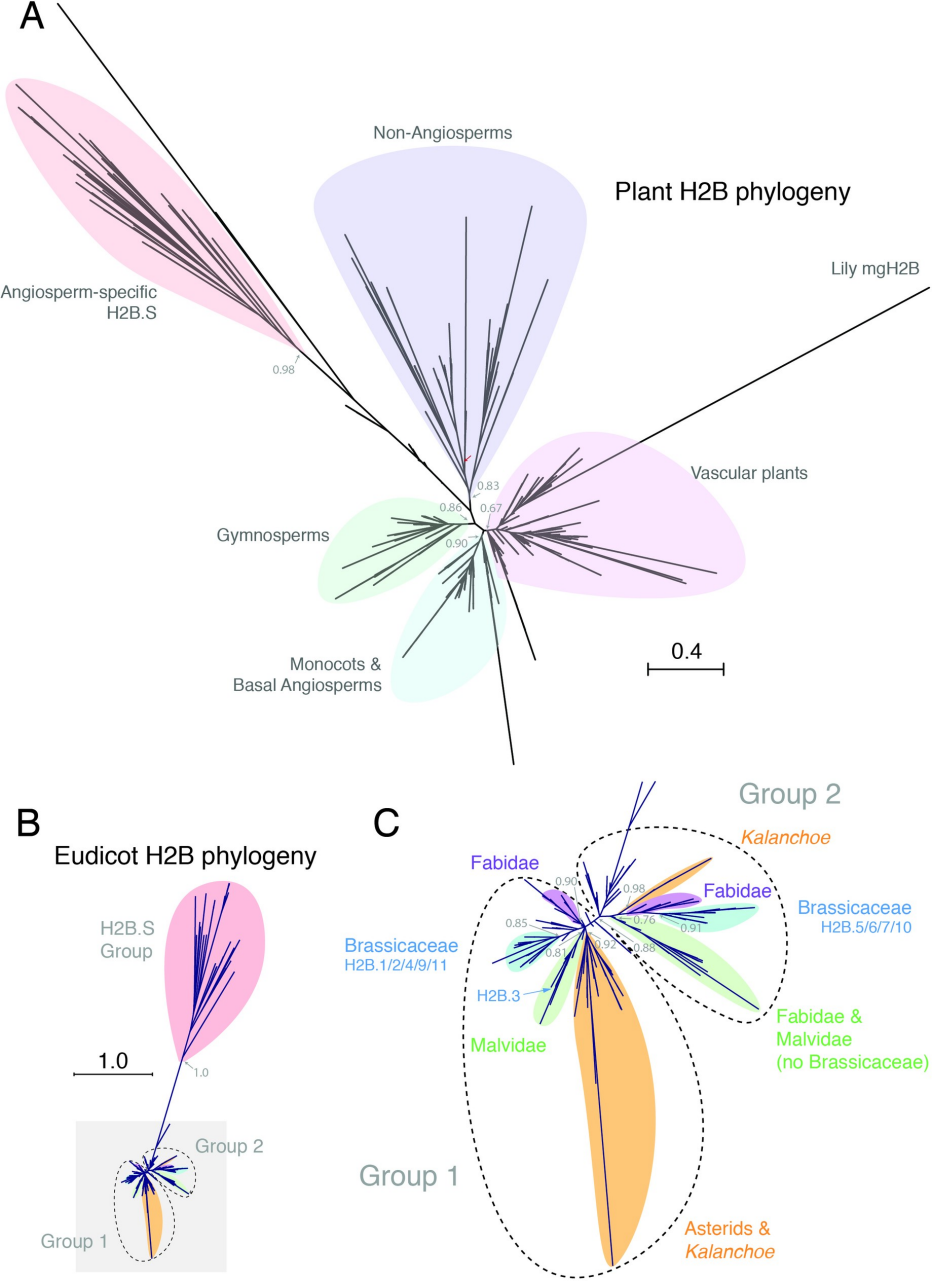
Phylogenetic analysis of the histone H2B family in the plant kingdom. (A) Maximum likelihood tree of H2Bs across the plant kingdom. Major clades are indicated by differently coloured shading. The non-angiosperm clade includes all sequences from Chlorophytes, Bryophytes, most Monilophytes, Charophytes, Lycophytes and several Gymnosperms. A highly divergent angiosperm-specific clade is also highlighted. Approximate likelihood ratio test values based on a Shimodaira-Hasegawa-like procedure are indicated on nodes to major clades. Scale bar indicates substitutions per site. Major plant groups are indicated with differently coloured shading. Red arrow indicates root as defined by the placement of the outgroup of Chlorophyte algae H2Bs. (B) Maximum likelihood tree of eudicot H2Bs. Three major clades are highlighted. Approximate likelihood ratio test value based on a Shimodaira-Hasegawa-like procedure is indicated on the H2B.S. Scale bar indicates substitutions per site. (C) Close-up view of two major groups of eudicot H2Bs highlighted with grey shading in the maximum likelihood tree shown in B. Family-specific clades are indicated with differently coloured shading. Approximate likelihood ratio test values based on a Shimodaira-Hasegawa-like procedure are indicated on nodes to major clades.

In plants, histone H2Bs have undergone significant sequence divergence and expansion in the number of encoding genes [5, 42]. In three distantly-related plant species–*Arabidopsis thaliana* (11), *Marchantia polymorpha* (6) and *Klebsormidium nitens* (3)–H2Bs vary substantially in the length and sequence of their N-terminal tails (S1A–S1C Fig) but not in the histone core domain (S2A and S2B Fig). This suggested that H2B proteins are highly diversified in plants, prompting us to assess evolutionary relationships across the plant kingdom. We thus performed a comprehensive phylogenetic analysis of H2B sequences from 77 plant genomes, ranging from Chlorophytes to Angiosperms (S1 Data) [5]. Although we observed distinct clades in our phylogenetic tree, most of these were formed by H2B sequences from each major plant lineage, such as gymnosperms and monocots (Fig 1A). A notable exception was a clade of highly divergent H2Bs present in all angiosperms except *Amborella trichopoda*, the most basal angiosperm species (Fig 1A). We failed to recover a eudicot clade of H2Bs, likely due to other vascular plant H2Bs contributing long branch attraction [43, 44], most notably Lily mgH2B (Fig 1A). We thus constructed a second phylogeny of only eudicot H2Bs (Fig 1B). We noted a prominent split among eudicot H2B sequences which, with the exception of H2Bs from Asterids, fell into three major phylogenetic groups—Group 1 containing *Arabidopsis* H2B.1/2/4/9/11, Group 2 containing *Arabidopsis* H2B.5/6/7/10 and the angiosperm-specific group containing *Arabidopsis* H2B.8 (Fig 1B and 1C). A dN/dS analysis of a representative subset of Eudicot H2Bs revealed significant relaxation of purifying selection for the H2B.S group (from 0.023 to 0.09), but not for Group 1 nor Group 2 (S1 Data). Our observations thus indicate extensive diversification of H2B proteins in the plant kingdom and point to prominent evolutionary constraints acting within each major clade. To gain more insight into this diversification, we focused our attention on the properties of the *Arabidopsis* H2B family.

### *Arabidopsis* H2B-encoding genes have diverse expression patterns

**Fig 2:**
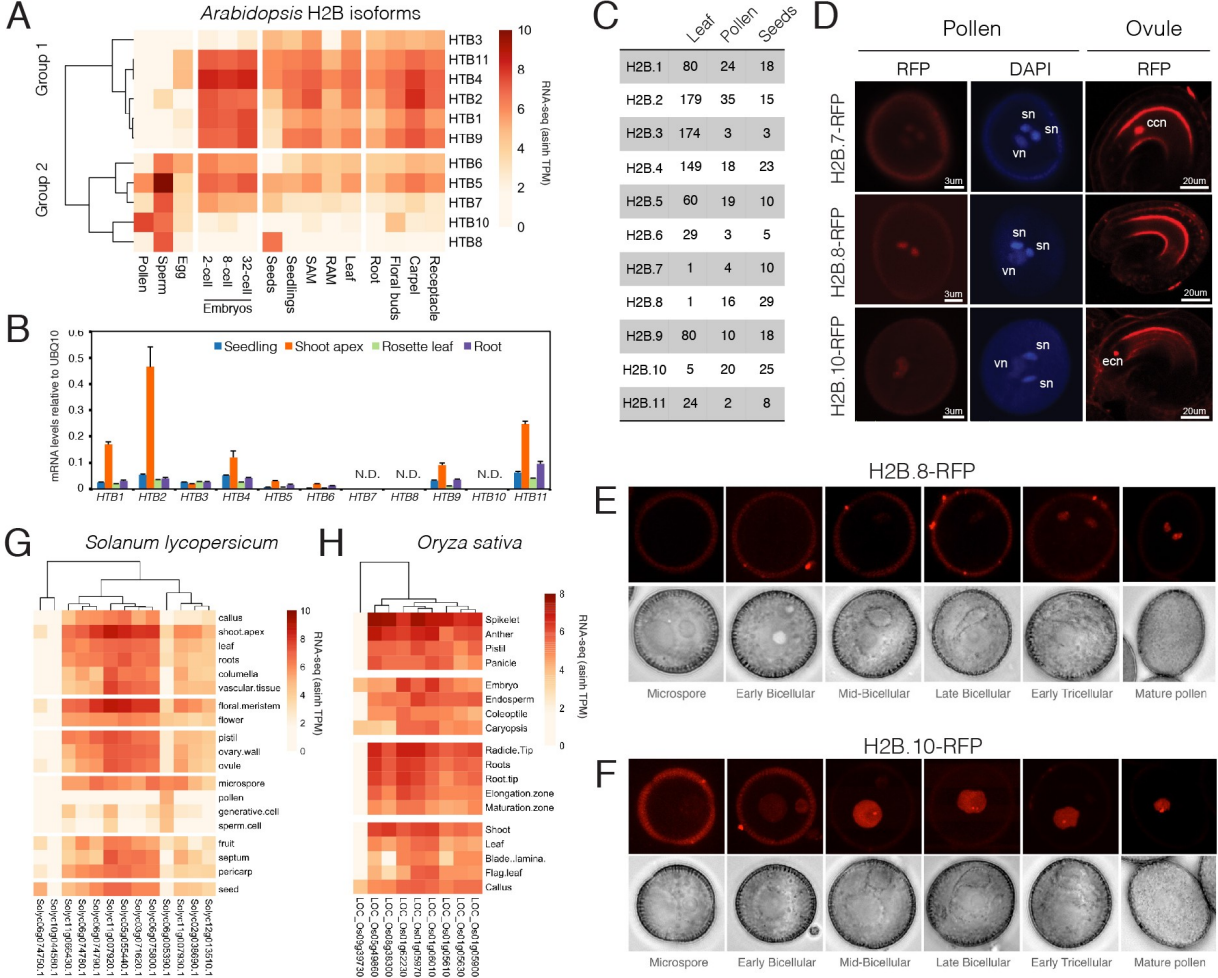
Developmental expression of *Arabidopsis* H2B-encoding genes. (A) Heat map showing expression levels of *Arabidopsis* H2B genes determined by RNA-seq. SAM: shoot apical meristem, RAM: root apical meristem. (B) Expression of *Arabidopsis* H2B genes measured by qRT-PCR in somatic tissues. Data represents relative expression levels normalized to *UBQ10*. Error bars represent the standard deviation from three biological replicates. N.D.—not detected. 10-day-old seedlings were used for RNA extraction with at least 20 seedlings per biological replicate. For shoot apex, rosette leaf and root, tissues were dissected from 3-week-old plants, with at least 30 plants used for dissection in each biological replicate. (C) Qualitative mass spectrometry analysis of *Arabidopsis* H2Bs. Spectral counts of unique peptides for *Arabidopsis* H2Bs in each sample are indicated. Data for leaves were obtained by immunoprecipitation of H3 after MNase digestion of leaf nuclei. Data for pollen and seeds were obtained from acid extracted histones. See Materials and Methods for details. (D) Expression of H2B.7-RFP, H2B.8-RFP, and H2B.10-RFP in pollen and ovules. DAPI staining is used to indicate two sperm nuclei (sn) and one vegetative nucleus (vn) in pollen, respectively. ccn: central cell nucleus, ecn: egg cell nucleus. (E,F) Expression of H2B.8-RFP (E) and H2B.10-RFP (F) during the different stages of pollen development. (G) Heat map showing expression levels of rice (*Solanum lycopersicum*) H2B genes in the RNA-seq datasets deposited in Genevestigator. (H) Heat map showing expression levels of rice (*Oryza sativa*) H2B genes in the RNA-seq datasets deposited in Genevestigator.

Aside from having divergent sequences, histone variants often differ from each other by distinct expression patterns [45]. In *Arabidopsis*, H2B genes clustered based on their expression in reproductive tissues (Fig 2A), which strikingly recapitulated the eudicot split we observed in our phylogenetic tree (Fig 1C). Notably, *HTB7*, *HTB8* and *HTB10* transcripts were highly abundant in pollen and/or sperm but were barely detectable or absent in somatic tissues like seedlings, leaf and root (Fig 2A and 2B). *HTB8* transcripts were also highly prominent in dry seeds (Fig 2A) while *HTB5* and *HTB6* transcripts were relatively enriched in sperm but still detectable in somatic tissues (Fig 2A and 2B). Mass spectrometry of histones isolated from leaf, pollen and dry seeds further confirmed these transcriptome profiles (Fig 2C). This suggests that, in addition to sperm-specific histone H3.10 [20, 21], *Arabidopsis* expresses a class of H2Bs with enriched or specific expression in sperm, pollen and seeds.

To explore the reproductive expression of H2Bs, we fused the genomic region containing the upstream promoter and coding region of each *Arabidopsis HTB* gene to an RFP reporter and examined RFP accumulation in male and female gametophytes. Except for H2B.3, which was not detected in any of the reproductive tissues we examined, we detected all H2B variants during reproductive growth (Table 1). In ovules, H2B.7-RFP was highly expressed in the central cell, while H2B.10-RFP was expressed in the egg cell (Fig 2D). In pollen, H2B.8-RFP and H2B.10-RFP were specifically detected in sperm or vegetative nuclei, respectively, while H2B.7-RFP was detected in both (Fig 2D). During pollen development, H2B.8-RFP expression was restricted solely to the sperm lineage (Fig 2E). In contrast, H2B.10-RFP expression was detected in microspores then remained largely restricted to the vegetative cell during pollen maturation (Fig 2F).

**Table 1:** Summary of pH2B:H2B-RFP signals in reproductive tissues.

	Sperm cell	Vegetative cell	Egg cell	Central cell
HTB1-RFP	D.	N.D.	N.D.	N.D.
HTB2-RFP	D.	N.D.	N.D.	N.D.
HTB3-RFP	N.D.	N.D.	N.D.	N.D.
HTB4-RFP	D.	D.	D.	D.
HTB5-RFP	D.	D.	D.	D.
HTB6-RFP	D.	D.	D.	D.
HTB7-RFP	D.	D.	N.D.	D.
HTB8-RFP	D.	N.D.	N.D.	N.D.
HTB9-RFP	D.	D.	D.	D.
HTB10-RFP	N.D.	D.	D.	N.D.
HTB11-RFP	D.	D.	D.	D.
D.: detected, N.D.: not detected				

We next assessed whether a subset of H2Bs were also restricted to male reproductive development in other eudicot species. In tomato, we again observed a clear bias in developmental expression (Fig 2G), with the expression of one H2B gene remaining largely restricted to male gametophytic cells (*Solyc06g005390*.*1*). Unlike eudicots, no obvious split was observed among histone H2B proteins in monocots (Fig 1A and 1B). Eight H2B genes in rice showed no obvious developmental bias and were equally expressed in both reproductive and somatic tissues (Fig 2H). However, two H2B genes in lily, also a monocot species (Fig 1A), have been shown to be preferentially expressed in pollen [46, 47], suggesting that this feature might have been lost in rice. These data show that histone H2Bs with preferential or specific expression in male reproductive cells are widespread in angiosperms and may indicate a common evolutionary origin.

### H2B.S variants form a clade of seed-specific histone H2Bs in plants

**Fig 3:**
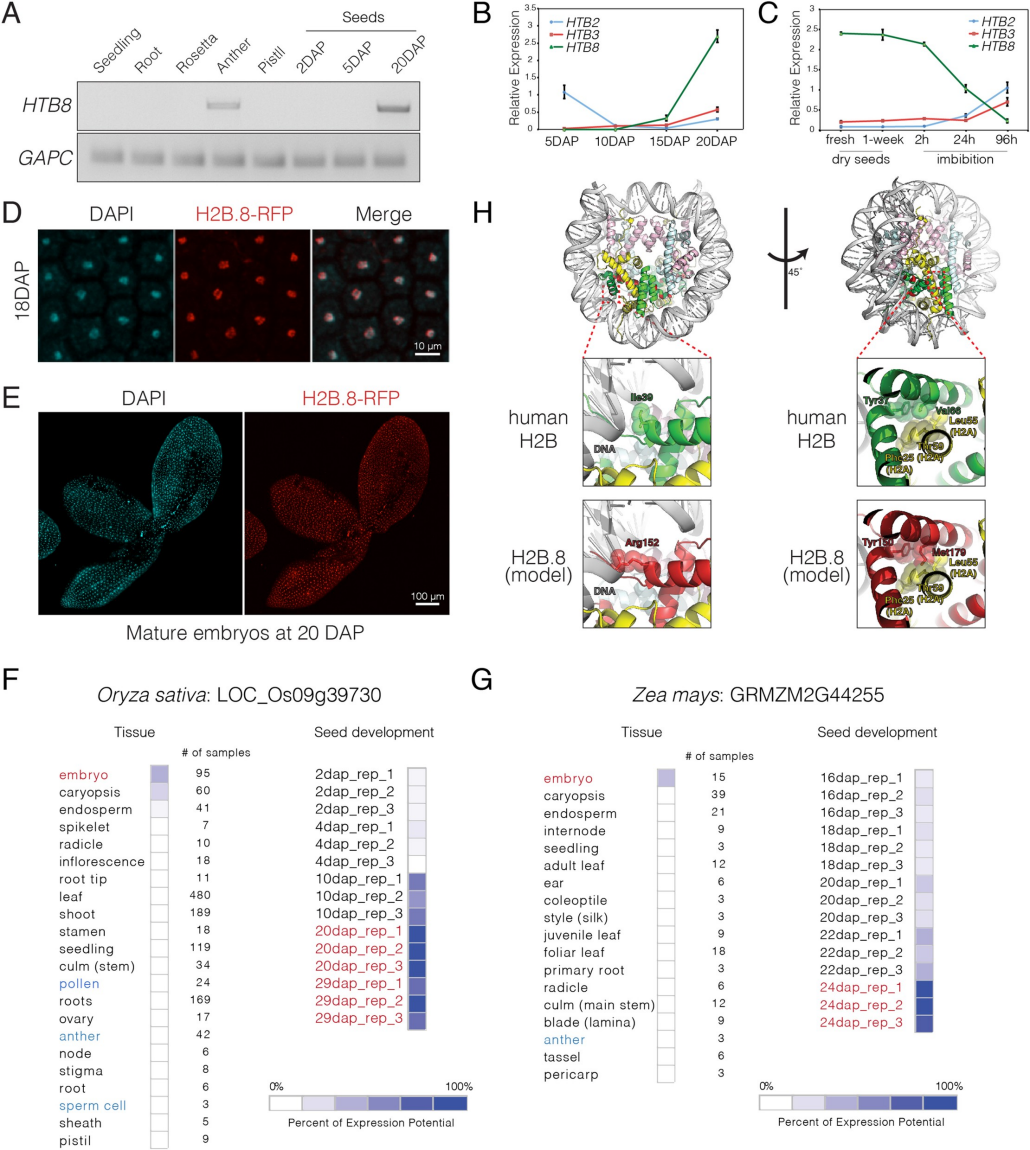
Expression analysis of H2B.S histone variants in *Arabidopsis*, rice and maize. (A) RT-PCR analysis of *HTB8* expression in *Arabidopsis* tissues. *GAPC* is served as an endogenous control. DAP: days after pollination. Seedling, root and rosettes were collected as described in Fig 2B. At least 150 mature anthers and 30 mature pistils were used for RNA extraction. Seeds were collected based on the days after pollination, with at least 500 seeds at 2DAP and 5DAP, and 250 seeds at 20DAP used for RNA extraction. (B) Expression of *HTB2*, *HTB3* and *HTB8* during seed maturation. Data represents relative expression levels normalized to *UBQ10*, error bars represent the standard deviation (SD) from three biological replicates. At least 500 seeds at 5DAP and 10DAP, and 250 seeds at 15DAP and 20DAP were used in each biological replicate. (C) Expression of *HTB2*, *HTB3*, and *HTB8* during seed storage and imbibition. Data represents relative expression levels normalized to *UBQ10*, error bars represent the SD from three biological replicates. At least 250 seeds at each time point were used in each biological replicate. (D) H2B.8-RFP expression in cotyledon nuclei of developing embryos at 18 DAP. DAPI staining indicates the position of nuclei. (E) H2B.8-RFP expression in a mature embryo alongside a corresponding DAPI stained image. (F-G) Expression of *HTB8* relatives in rice (*Oryza sativa*) and maize (*Zea mays*) in selected tissues and different stages of seed development, respectively. Data represents microarray transcriptomes deposited in Genevestigator. (H) Modelled structure of an H2B.8-containing nucleosome with a close-up view of the predicted location of highly conserved amino acids in H2B.S orthologs, which are highlighted in red. Yellow indicates histone H2A, green indicates histone H2B, pink indicates histone H3 and blue indicates histone H4. The position of the ten highly conserved H2B.S amino acids within the nucleosome are marked in red.

We also noted that, like *Arabidopsis HTB8* (Fig 2A), both tomato (*Solyc06g074750*.*1*) and rice (*LOC_Os09g39730*) had histone H2Bs with seed-specific expression (Fig 2G and 2H), which clustered together with other highly divergent H2Bs from eudicots and monocots (Fig 1B and 1C). This indicated the presence of a seed-specific clade of histone H2B proteins in flowering plants. Further characterization of *HTB8* expression during seed development revealed that transcripts only became detectable in mature seeds 20 days after pollination (DAP) (Fig 3A). Consistently, qRT-PCR analysis revealed a dramatic increase in *HTB8* transcripts during the late stages of seed development around 15 DAP (Fig 3B). Once dry seeds were formed, *HTB8* transcript levels remained stable during storage but dropped drastically during imbibition and subsequent germination (Fig 3C). Analysis of the H2B.8-RFP reporter line revealed accumulation in embryo nuclei at 18 DAP (Fig 3D) around the time that chromatin begins to compact [48]. At the mature seed stage 20 DAP, nuclear H2B.8-RFP expression was observed in all nuclei of the mature embryo (Fig 3E). These results show that *Arabidopsis* H2B.8 is specifically expressed in sperm and mature embryos, suggesting an adaptive function in cell types that become desiccated, quiescent or show higher degrees of chromatin compaction.

We sought to determine whether the expression pattern of H2B.8-related genes is conserved in other angiosperms. Our analysis in rice already revealed a seed-specific H2B (Fig 2H), which incidentally formed part of the same clade with another ortholog in maize (S3B Fig). We thus analysed the expression of these H2B.8-related genes using rice and maize transcriptomes deposited in Genevestigator [49]. Strikingly, we found that both H2Bs were also highly and specifically expressed in rice and maize embryos, with a clearly biased enrichment in maturing dry seeds (Fig 3F and 3G), suggesting an evolutionary conserved expression pattern. Interestingly, unlike *Arabidopsis HTB8*, expression in anthers, pollen or sperm cells was not evident neither in rice nor maize (Fig 3F and 3G). Thus, H2B.8 forms part of a distinct group of seed specific H2B variants in plants, with *Arabidopsis HTB8* likely having acquired sperm expression during dicot evolution.

The angiosperm-specific clade of H2B.8 orthologs were characterized by conserved substitutions in the histone core, slightly extended C-terminal tails and greatly expanded N-terminal tails with a KVVXETV motif (S3A Fig). To examine where the histone core substitutions might lie within the nucleosome, we modelled an H2B.8-containing nucleosome using the published human nucleosome structure as a template (Fig 3H) [50]. Eight out of the ten amino acids in human H2B were conserved in H2B.9 but not in H2B.8 (S2C Fig). These H2B.8-specific amino acids were all orientated towards the inner core of the nucleosome and faced histone H2A, H4 or H2B.8 itself (Fig 3H). Intriguingly, two H2B.8 residues, Arg152 and Met179, were highly conserved among H2B.8 orthologs (S3A Fig) and were positioned in such a way that they might contribute to stronger interactions with DNA and histone H2A, respectively (Fig 3H). These observations suggest that sequence divergence in H2B.8 impact intra-nucleosomal interactions to potentially alter nucleosome structure and/or stability.

We propose to define H2B.S as a new class of histone variants—comprising of *Arabidopsis* H2B.8 and its related orthologs—as they have distinct phylogenetic origins, share characteristic substitutions and have seed-specific expression. Despite forming a distinct angiosperm-specific clade, H2B.S variants show a high degree of variation, even amongst closely related genera (S3B Fig), with relaxed purifying selection indicating rapid evolution and potential species-specific adaptation. Other highly divergent sequences from Streptophyte algae and gymnosperms form a grade basal to H2B.S variants (S3B Fig), suggesting a deeper evolutionary origin. However, the lack of conserved substitutions (S3A Fig) and similar sequences in bryophytes, lycophytes and ferns led us to exclude them as H2B.S-related variants at this time.

### Chromatin localization of *Arabidopsis* somatic H2Bs

**Fig 4:**
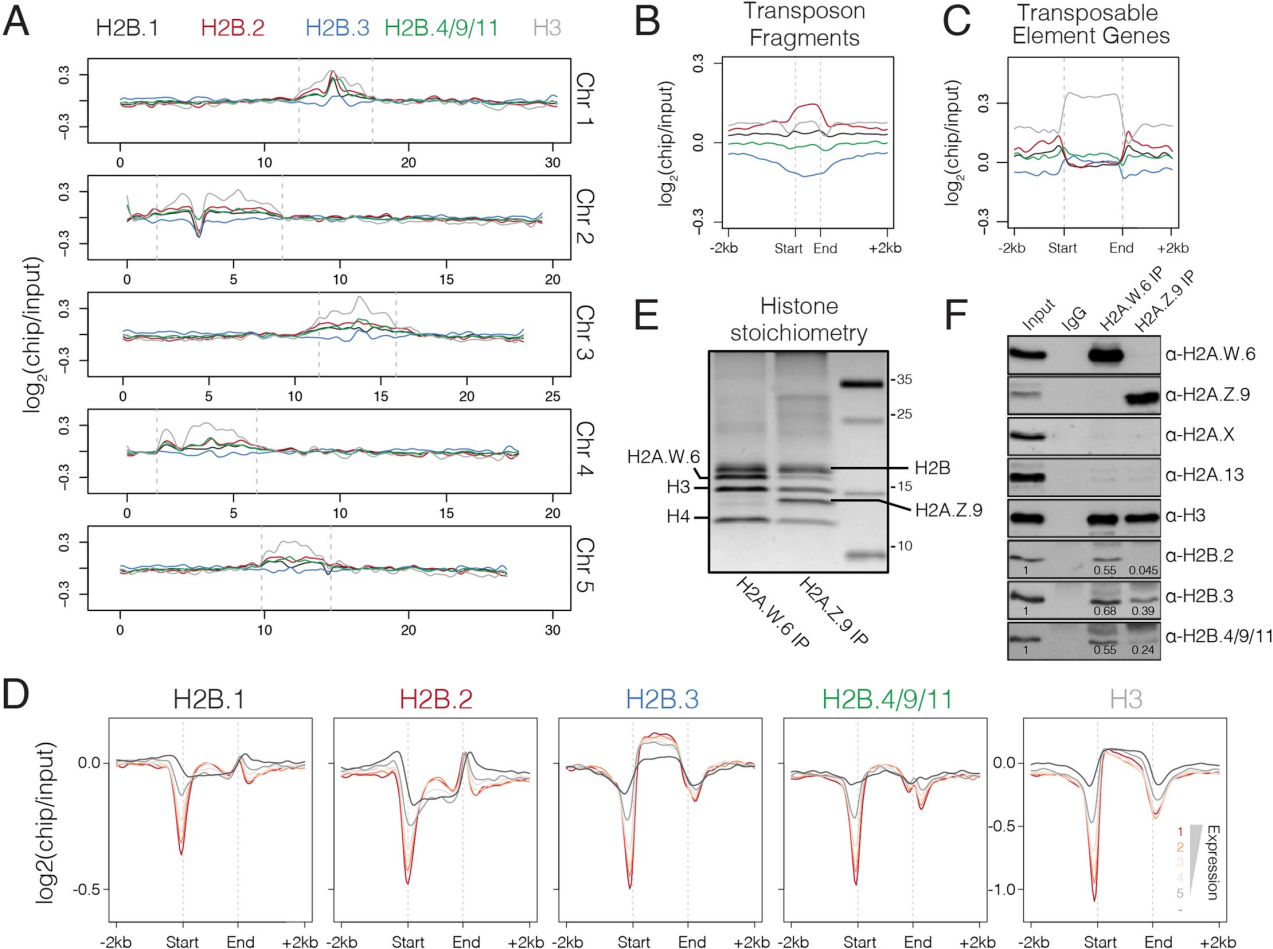
Genomic distribution of somatic *Arabidopsis* H2Bs. (A) Chromosomal distribution of somatic H2Bs alongside histone H3 over each of the five *Arabidopsis* chromosomes calculated in 100 kb bins. Plotted is the ChIP-seq log_2_ enrichment relative to input. Pericentromeric regions are indicated by dashed grey lines. (B-C) Distribution of somatic H2Bs and histone H3 over transposon fragments (B) and transposable element genes (C). Plotted is the ChIP-seq log_2_ enrichment relative to input. (D) Distribution of somatic H2Bs and histone H3 over genes grouped by their level of expression in *Arabidopsis* seedlings. Each group is colour coded using the scheme shown with non-expressed genes shown in dark grey. (E) Silver stained gel of immunoprecipitated H2A.W.6 and H2A.Z.9 mono-nucleosomes confirms histone stoichiometry. Identities of protein bands are indicated. (F) Western blotting analysis of samples in panel E with indicated antibodies. Enrichment of H2Bs was calculated relative to input and normalized to H3 in each sample.

To investigate whether *Arabidopsis* H2B variants expressed in somatic cells decorate specific chromatin features, we developed antibodies that specifically recognize H2B.1, H2B.2, H2B.3, and the clade comprising H2B.4, H2B.9 and H2B.11 (S4A Fig). The specificity of these antibodies was validated using corresponding knock-out mutants and RFP reporter lines (S4B–S4E Fig). We generated genomic profiles of each H2B in seedlings using chromatin immunoprecipitation followed by high-throughput sequencing (ChIP-seq). The somatic H2Bs localized at both euchromatic and constitutive heterochromatic regions, which were marked by low and high histone H3 enrichment, respectively (Fig 4A) [51]. Compared with other H2B variants, H2B.3 was relatively depleted from constitutive heterochromatin while H2B.2 was relatively enriched (Fig 4A). A similar pattern was observed over transposon fragments (Fig 4B), which represent short remnants of transposable element (TE) insertions that accumulate within heterochromatic regions.

We also assessed H2B enrichment over TE genes, which represent long and intact TEs that, like protein-coding genes, have the potential to be transcribed (Fig 4C). H2B.3 was strongly depleted from the flanking regulatory region of TE genes while H2B.2 was strongly enriched (Fig 4C). This same pattern was also evident over protein-coding genes (S5A Fig). H2B.2, and to a lesser degree H2B.1, had the highest relative enrichment in the regions upstream and downstream of un-transcribed protein coding genes (Fig 4D). In contrast, gene bodies were occupied primarily by H2B.3 and H2B.4/9/11 (Fig 4D and S5A Fig). While H2B.4/9/11 showed no obvious correlation with gene expression, we observed a somewhat positive correlation between H2B.3 enrichment and transcript levels (Fig 4D). Thus, H2B.3 deposition is enriched over the gene body of protein coding genes, while H2B.2 prefers transcriptionally inactive regions of the genome.

We also assessed the association of H2Bs with nucleosomes containing either H2A.Z.9 or H2A.W.6 (Fig 4E), which represent euchromatin and constitutive heterochromatin, respectively. We detected similar enrichment of H2B.2, H2B.3 and H2B.4/9/11 in H2A.W.6-containing nucleosomes (Fig 4F). In contrast, H2B.3 was the most enriched in H2A.Z.9 nucleosomes while H2B.2 was hardly detectable (Fig 4F). These results demonstrate how specific somatic *Arabidopsis* H2Bs are preferentially deposited at active or inactive regions of the genome.

### *Arabidopsis* H2B.3 has the hallmarks of a replacement histone variant

**Fig 5:**
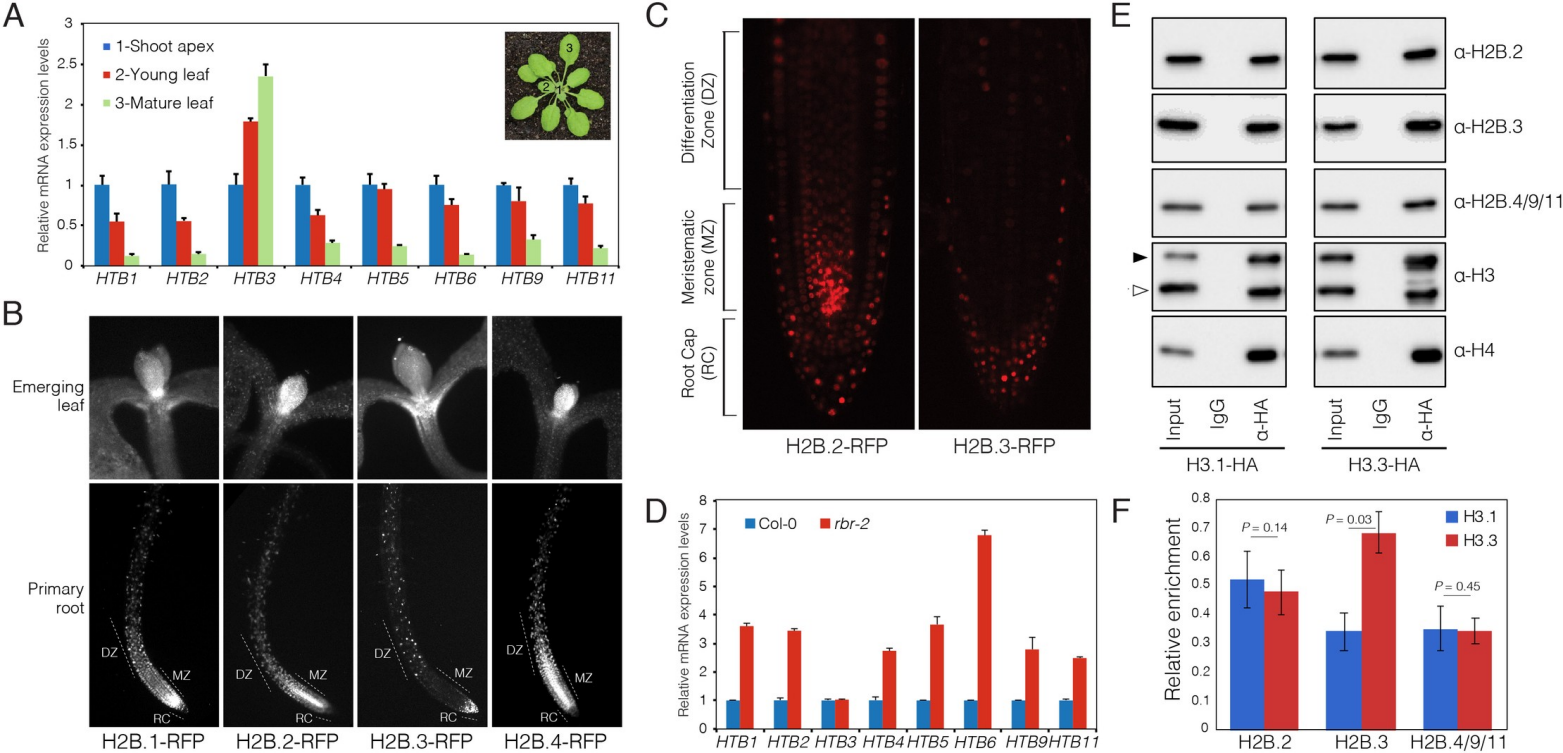
*Arabidopsis* H2B.3 is preferentially expressed in non-dividing cells. (A) Relative expression of *Arabidopsis* somatic H2Bs in the shoot apex, young leaf and mature leaf, which represent tissue with decreasing mitotic activity from shoot apex to mature leaf. Values represent fold-changes relative to expression in the shoot apex. Error bars represent the standard deviation (SD) from three biological replicates. Leaves and shoot apex were dissected from 4-week-old plants, with at least 30 plants used for dissection of each biological replicate. (B) Expression of H2B-RFP fusion proteins in emerging leaf and primary root tissue. DZ = differentiation zone, MZ = meristematic zone, RC = root cap. (C) Comparison of H2B.2-RFP and H2B.3-RFP expression in the root tip illustrates the absence of H2B.3 from the MZ but presence in non-dividing cells of the RC. (D) Relative expression of *Arabidopsis* somatic H2Bs in the *rbr-2* mutant characterized by increased mitotic activity. Values represent fold-changes relative to expression in wild type Col-0. Error bars represent the SD from three biological replicates. (E) HA-tagged H3.1 and H3.3 were immunopurified and analysed for the presence of H2Bs. Black and white triangles on the H3 blot indicate bands of HA-tagged and endogenous histone H3, respectively. (F) Quantification of H2Bs precipitated with H3.1 and H3.3. Data represents the mean ± standard deviation of three independent experiments normalized to the total H3 signal. Statistical significance was assessed using a one-tailed paired Student’s t-test.

Some histone variants, like histone H3.3, are typically expressed independent of DNA replication and act as replacement histones during processes like transcription [52]. We noted that the distribution of H2B.3 over genes mirrored that of histone H3.3 [12, 14], suggesting that H2B.3 might function as a replacement histone. To explore this, we analysed relative mRNA expression of the somatic H2B variants in young leaf and mature leaf compared to the shoot apex (Fig 5A). The shoot apex typically contains a high proportion of actively dividing cells, while mature leaves contain differentiated non-dividing cells. Most of the somatic H2B genes had enriched expression in the shoot apex compared to the other tissues (Fig 5A), suggesting that they are expressed and incorporated into nucleosomes of proliferating cells. In contrast, *HTB3* expression levels were enriched in mature leaves compared to young leaves and shoot apex (Fig 5A). A similar pattern was also observed in transcriptome profiles of *Arabidopsis* suspension cultures (Table 2) [53], where H2B.3 had non-periodic expression during the cell cycle, further suggesting that H2B.3 expression might be independent of cell division. This was largely confirmed when we examined H2B protein levels using RFP reporter lines. H2B.1-RFP, H2B.2-RFP and H2B.4-RFP were highly expressed in young emerging leaves and the meristematic zone of the root tip (Fig 5B and 5C), which contain a high number of actively dividing cells. In contrast, H2B.3-RFP signals were deficient in both tissues but apparent in differentiated cells of the differentiation zone and root cap (Fig 5B and 5C).

**Table 2:** Periodicity of somatic H2Bs in *Arabidopsis* suspension cultures.

Gene name	AGI	Peaktime	Rank
HTB1	AT1G07790	non-periodic	411
HTB2	AT5G22880	S	291
HTB3	AT2G28720	non-periodic	3805
HTB4	AT5G59910	S	314
HTB5	AT2G37470	S	83
HTB6	AT3G53650	S	223
HTB9	AT3G45980	S	110
HTB11	AT3G46030	S	75
Data from Cyclebase 3.0 (REF)			

To test the link between H2B.3 and the cell cycle further, we analysed the expression of the somatic H2Bs in a mutant background of *RETINOBLASTOMA RELATED (RBR)*, a plant homolog of the tumour-suppressor Retinoblastoma, which acts as a cell cycle repressor that prevents transition from G1 to S-phase [54]. Thus, the proportion of actively dividing cells is increased in *Arabidopsis rbr* mutants [55]. Unlike the other somatic H2Bs, which had increased expression in *rbr*, expression of *HTB3* remained unchanged (Fig 5D).

If H2B.3 is deposited primarily in non-dividing cells, we predicted that it would be enriched in nucleosomes containing H3.3. We thus analysed whether nucleosomes containing H2B.2, H2B.3 and H2B.4/9/11 were enriched for H3.1 or H3.3. We immunopurified mono-nucleosomes using HA-agarose beads from leaves of transgenic *Arabidopsis* plants expressing C-terminal HA-tagged H3.1 (*pHTR13*:*HTR13-HA* in *htr13*) and H3.3 (*pHTR5*:*HTR5-HA* in *htr5*) [10]. Western blots revealed that all three H2Bs were detected in immunoprecipitated H3.1 and H3.3 nucleosomes (Fig 5E and S5B Fig). However, while H2B.2 and H2B.4/9/11 were present at similar levels with H3.1 and H3.3, H2B.3 was significantly enriched in nucleosomes containing H3.3 (Fig 5E and 5F and S5B Fig). We conclude that H2B.3 might act as a replacement histone in non-dividing cells, while the other four somatic H2Bs are primarily expressed and deposited in proliferating cells.

## Discussion

Our study has highlighted the evolutionary diversification and expansion of H2B proteins in the plant kingdom. Amongst flowering plants, eudicots experienced the highest degree of divergence among H2B genes. This divergence appears to have been driven by preferential expression during gametogenesis, including three genes in *Arabidopsis* (H2B.7/8/10) and at least one in tomato. Male germline-specific H2Bs are also present in the monocot lily [46], indicating that tissue-specific H2Bs are widespread among flowering plants and possibly share a common origin in angiosperms, constituting a potentially novel class of H2B variants. In mammals, testis-specific TH2B variants are distinguished by distinct amino acid residues in their N-terminal tail and histone core domain that might affect nucleosome stability [24, 35]. Similarly, much of the variation in the sperm-enriched *Arabidopsis* H2Bs also lies in the N-terminal tail and histone core domain, suggesting a potential for unique post-translational modifications and nucleosome properties in these cell types. *Marchantia polymorpha* encodes the divergent histone MpH2B.5 although it does not appear to be expressed during male gametogenesis, which is characterised by the replacement of histones with protamines during sperm maturation [56]. Unlike their ancestors, flowering plants lost sperm motility and do not package sperm chromatin with protamines [17]. Deposition of these sperm-enriched histone H2Bs might influence chromatin processes during male gametogenesis. The link with male gametogenesis is proposed to be a driving force in the evolution and diversity of histone variants in eukaryotes [57]. This is also illustrated by testis-expressed H2As in primates [34] and pollen-expressed H3 variants in flowering plants [17]. Further functional validation of knock-out mutants of gametophytic H2Bs and chromatin profiling in pollen will be required to substantiate their proposed role in reproductive biology.

Our analyses have revealed a new class of seed-specific histone variants in flowering plants, H2B.S. This class of H2B variants includes *Arabidopsis* H2B.8, which is also expressed in *Arabidopsis* sperm, although the lack of sperm expression for H2B.S members in monocots suggests that this feature might have been acquired during dicot evolution. The lysine residue in the C-terminal tail that is subjected to monoubiquitination in other H2Bs is substituted to asparagine in H2B.8. H2B monoubiquitination (H2Bub1) acts as an active histone modification that promotes transcription [58–60]. Interestingly, H2B.8 strongly accumulates in sperm and mature embryos, where chromatin is relatively compact and less transcriptionally active. Structural modelling of an H2B.8-containing nucleosome revealed that highly conserved amino acid residues in H2B.S are orientated at key positions within the nucleosome, with two in particular having the potential to increase nucleosome stability and DNA binding. Future studies will be needed to clearly decipher how H2B.S deposition might alter chromatin properties and influence the drastic structural chromatin transitions occurring during spermatogenesis and seed maturation in flowering plants.

In addition, we also identified H2B.3 as a likely candidate for a replacement H2B variant. Replacement histone variants, such as histone H3.3 and H2A.Z, are characterized by their sequence divergence, DNA replication-independent expression patterns and specific genomic localization [12, 14, 31]. We have shown that *HTB3*, unlike other H2Bs, is preferentially expressed in tissues enriched in non-proliferating cells, suggesting that it is deposited as a replacement histone. Consistently, H2B.3 shows a distribution similar to that of H3.3 and H2A.Z in plants, which are deposited in a DNA replication-independent manner [61]. The links between H2B.3 expression and deposition during the cell cycle thus warrant further investigation. While H2A.Z shows specialized sequence characteristics that are conserved during evolution [35], H2B.3 has no prominent sequence differences that distinguish it as a distinct H2B in other closely-related species in a manner similar to H3.1 and H3.3 diversification [5, 62]. This suggests that the functional diversification of histone H2B might have arisen from differing regulation that is independent of the cell cycle. H2B.3 thus potentially represents a new class of histone variant although further analyses and identification of replacement H2Bs in other species are required to confirm this. Because the somatic H2Bs do not exhibit conserved prominent sequence characteristics in other species or individually cause any strong effects on development, it remains difficult to classify them as distinct histone variants at this point. Nevertheless, genomic profiling and mass spectrometry has revealed surprising differences in the association of H2Bs within euchromatin and constitutive heterochromatin. This suggests functional specialization among members of the H2B family and that specific deposition mechanisms might exist in flowering plants to generate this diversity.

## Methods

### Plant materials and growth conditions

T-DNA insertion mutants of *Arabidopsis* H2B coding genes *htb1* (RATM15-1815-1_G), *htb2* (SALK_008776), *htb3* (SALK_063387), *htb4* (SALK_036467) and *htb9* (SAIL_839_B06) were obtained from Arabidopsis Biological Resource Center (ABRC). The *rbr-2* mutant was described previously [63]. Plants were grown under long day (16 h light/8 h dark) or short day (8 h light/16 h dark) conditions at 22°C.

### Phylogenetic analyses

*Arabidopsis thaliana* (AT3G53650), *Marchantia polymorpha* (Mapoly0053s0016) and *Klebsormidium nitens* (kfl00099_g20_v1.1) H2B protein sequences were used in BLAST searches of Phytozome (https://phytozome.jgi.doe.gov/pz/portal.html), Fernbase (https://www.fernbase.org/), Orcae (https://bioinformatics.psb.ugent.be/orcae/overview/Chbra), waterlilyPond (http://waterlily.eplant.org/), Congenie (http://congenie.org/), Plaza (https://bioinformatics.psb.ugent.be/plaza/versions/gymno-plaza/blast/index) and Cotton databases (http://cotton.hzau.edu.cn/EN/download.php). Hits with e-scores less than 10^−2^ were retrieved. A rough alignment was made with CLC Genomics Workbench 11.0 and sequences with insertions or deletions in the core histone domain were removed. The remaining sequences were aligned with MAFFT v7.427 [64] using default settings with minor manual correction. All sequences and alignments can be found in Supplemental Data 1. Substitution models for phylogenetic analyses were chosen using ProtTest v3.4.2 [65]. JTT+I+G+F was used for both plant and eudicot phylogenies, with an alpha value of 0.900 and 0.912 and a p-invariant value of 0.005 and 0.040, respectively. Phylogenies were constructed with PhyML v3.3 [66] with the following settings: -b -4 -d aa -m JTT -a <alpha> -v <p-invariant> -f e–r_seed 1562932227 –no_memory_check, where <alpha> and <p-invariant> are the appropriate values determined by ProtTest. Branch supports were calculated using approximate likelihood ratio tests based on a Shimodaira-Hasegawa-like procedure, where values greater than 0.9 indicate strong support [67]. Full trees can be found in Supplemental Data 1.

A subset of Eudicot H2Bs representing major lineages were selected for the analyses on selection pressures. Amino acid alignments and tree were generated as above, except the LG+G model was used for tree construction, with the whole settings as follows: -b -4 -d aa -m LG -f m -v 0 -a e -c 4 -o tlr—r_seed 1562932227—no_memory_check. The coding sequence alignment was generated using PAL2NAL [68] from the amino acid alignment and trees were manually labelled for H2B.S, Group 1 and Group 2 branches. dN/dS analyses were performed in PAML 4.9 [69] for both branch and branch-site tests. For branch tests, the following settings were used: seqtype = 1, CodonFreq = 0, clock = 0. For the M0 test, model = 0 and NSsites = 0. For branch tests, model = 2 and NSsites = 0. For branch-sites tests, model = 2, NSsites = 2, and omega was either set to 1 or estimated. Likelihood ratio tests were performed between branch models and M0 or between branch-site models and models where omega is fixed at 1 with results shown in Supplemental Data 1.

All scripts pertaining to phylogenetic analyses can be found at https://github.com/seanmontgomery/H2B.

### Plasmid construction and generation of H2B-RFP transgenic plants

For each of H2B-RFP marker line, we used a genomic fragment containing the complete upstream promoter sequence up to the nearest neighboring gene and the coding region without a stop codon. These were amplified from WT genomic DNA with attB1-H2B-F and attB2-H2B-R primers (S2 Data) using the KOD-plus-PCR kit (Toyobo). PCR fragments were cloned directionally into pDONR/Zeo using BP clonase (Invitrogen) to generate a pDONR/Zeo-H2B entry vector. Recombination LR reactions were performed between a *p*Alli-2 destination vector [70] and the pDONR/Zeo-H2B vector to generate recombined destination plasmids with each H2B gene fused to the N-terminus of red fluorescent protein (pH2B::H2B-RFP). These constructs were transformed into WT (Col-0 accession) plants via *Agrobacterium tumefaciens*–mediated floral dip method. Primary transformants were selected based on GFP positive signal in seeds.

### Cytological analysis of fluorescent marker lines

Analysis of H2B.8-RFP and H2B.10-RFP marker lines during pollen development was performed as described previously [71]. Spores were released from staged anthers into 0.3 M mannitol and imaged immediately. Developmental stages were determined using brightfield microscopy. Microspores were distinguished by a single nucleus with a prominent nucleolus. Early bicellular stages were identified by the prominent round membrane surrounding the generative cell. At the late bicellular stage, the generative cell elongates to form a distinct tail. DAPI-stained pollen grain images were taken by washing out pollen from mature open flowers into DAPI staining solution (0.1 M sodium phosphate pH 7.0, 1 mM EDTA, 0.1% Triton X-100, 0.4 μg/ml DAPI). Analysis of H2B.7-RFP, H2B.8-RFP and H2B.10-RFP marker lines in unfertilized ovules was performed as described previously [20]. Analysis of H2B.8-RFP marker lines in mature seeds was performed as described previously [48]. The first open flowers were marked to track seeds from the 1^st^ day of pollination. Whole mature embryos were manually isolated from intact seeds using dissection needles under a stereo microscope into DAPI staining solution and imaged immediately. Analysis of H2B.1-RFP, H2B.2-RFP, H2B.3-RFP and H2B.4-RFP marker lines in roots and emerging leaf were taken by mounting seedlings in distilled water prior to imaging. All images were taken using a ZEISS LSM 700 laser scanning confocal microscope.

### Nuclei isolation, MNase digestion, immunoprecipitation, SDS-PAGE and western blotting

For data presented in Fig 5E, nuclei were isolated from three-week old leaves from transgenic lines expressing *HTR5* (H3.1) and *HTR13* (H3.3) under their native promoters fused to a C-terminal HA tag in the respective mutant background [10]. Three-week old wild type leaves were used for nuclei isolation and immunoprecipitation of H2A.W.6 and H2A.Z.9 mononucleosomes (Fig 4E and 4F). MNase digestion and immunoprecipitation were performed as previously described [30]. Isolated nuclei were washed twice in 10 ml of N buffer (15 mM Tris-HCl pH 7.5, 60 mM KCl, 15 mM NaCl, 5 mM MgCl_2_, 1 mM CaCl_2_, 250 mM sucrose, 1 mM DTT, 10 mM ß-glycerophosphate) supplemented with protease and phosphatase inhibitors (Roche). After spinning for 5 min at 1,800 × *g* at 4°C, nuclei were re-suspended in N buffer to a volume of 1 ml. 20 μl of MNase (0.1 u/μl) (SigmaAldrich) were added to each tube and incubated for 20 min at 37°C; during the incubation, nuclei were mixed 4 times by inverting the tubes. MNase digestion was stopped on ice by the addition of 110 μl of MNase stop solution (100 mM EDTA, 100 mM EGTA). Nuclei were lysed by the addition of 110 μl of 5 M NaCl (final concentration of 500 mM NaCl). The suspension was mixed by inverting the tubes and they were then kept on ice for 15 min. Extracts were cleared by centrifugation for 10 min at 20,000 × *g* at 4°C. Supernatants were collected and centrifuged again as above and finally all extracts were mixed into one tube. For each immunoprecipitation, extract equivalent to 4 g of leaf material was used, usually in a volume of 1 ml. To control MNase digestion efficiency, 150 μl of the extract were kept for DNA extraction. Antibodies, including non-specific IgG from rabbit, were bound to protein A magnetic beads (GE Healthcare) and then incubated with MNase extracts over night at 4°C. Beads were washed two times with N buffer without sucrose containing 300 mM NaCl, followed by three washes with N buffer without sucrose containing 500 mM NaCl and one wash with N buffer without sucrose containing 150 mM NaCl. Anti-HA agarose beads (Roche) were used for immunoprecipitation of HA-tagged H3.1 and H3.3.

Proteins were resolved on 15% SDS-PAGE, transferred to a Protran nitrocellulose membrane (GE Healthcare) and analysed by Western blotting using standard procedures. The blots were developed with an enhanced chemiluminescence kit (Thermo Fischer Scientific) and signals documented by a ChemiDoc instrument (BioRad). All primary histone variant-specific antibodies were used at 1 μg/ml. H3 (Abcam 1791) and H4 (Abcam 10158) specific antibodies were used at a 1:2,000 dilution. Secondary antibodies, goat anti-rabbit IgG (BioRad) and goat anti-mouse IgG (BioRad) were used at 1:10,000 dilution. Antibodies against H2A.W.6, H2A.13, H2A.X [30, 31].

### Nano LC-MS/MS analysis

Histone bands corresponding to H2Bs were excised from silver stained gels of immunopurified H3.1. and H3.3, reduced, alkylated, digested in-gel with trypsin, LysC, and subtilisin, and processed for MS. Histones from dry seeds and pollen were acid extracted and TCA precipitated followed by propionylation and trypsin digestion. The nano HPLC system used was a Dionex UltiMate 3000 HPLC RSLC (Thermo Fisher Scientific, Amsterdam, Netherlands) coupled to a Q Exactive mass spectrometer (Thermo Fisher Scientific, Bremen, Germany), equipped with a Proxeon nanospray source (Thermo Fisher Scientific, Odense, Denmark). Peptides were loaded onto a trap column (Thermo Fisher Scientific, Amsterdam, Netherlands, PepMap C18, 5 mm × 300 μm ID, 5 μm particles, 100 Å pore size) at a flow rate of 25 μl/min using 0.1% TFA as the mobile phase. After 10 min, the trap column was switched in line with the analytical column (Thermo Fisher Scientific, Amsterdam, Netherlands, PepMap C18, 500 mm × 75 μm ID, 2 μm, 100 Å). Peptides were eluted using a flow rate of 230 nl/min, and a binary 2-hour gradient. The gradient starts with the mobile phases: 98% solution A (water/formic acid, 99.9/0.1, v/v) and 2% solution B (water/acetonitrile/formic acid, 19.92/80/0.08, v/v/v), increasing to 35% of solution B over the next 120 min, followed by a gradient in 5 min to 90% of solution B, stays there for 5 min and decreases within 5 min back to the gradient 98% of solution A and 2% of solution B for equilibration at 30°C.

The Q Exactive HF mass spectrometer was operated in data-dependent mode, using a full scan (m/z range 380–1500, nominal resolution of 60,000, target value 1E6) followed by MS/MS scans of the 10 most abundant ions. MS/MS spectra were acquired using normalized collision energy of 27%, isolation width of 1.4 m/z, resolution of 30,000 and the target value was set to 1E5. Precursor ions selected for fragmentation (exclude charge state 1, 7, 8, >8) were put on a dynamic exclusion list for 40 s. Additionally, the minimum AGC target was set to 5E3 and intensity threshold was calculated to be 4.8E4. The peptide match feature was set to preferred and the exclude isotopes feature was enabled. For peptide identification, the RAW files were loaded into Proteome Discoverer (version 2.1.0.81, Thermo Scientific). All hereby created MS/MS spectra were searched using MSAmanda v2.1.5.8733 [72] against *Arabidopsis thaliana* H2B histones. The following search parameters were used: Beta-methylthiolation on cysteine was set as a fixed modification, oxidation on methionine, deamidation on asparagine and glutamine, acetylation on lysine, phosphorylation on serine, threonine and tyrosine, methylation and di-methylation on lysine and threonine, tri-methylation on lysine and ubiquitylation on lysine were set as variable modifications. Monoisotopic masses were searched within unrestricted protein masses for tryptic enzymatic specificity. The peptide mass tolerance was set to ±5 ppm and the fragment mass tolerance to ±15 ppm. The maximal number of missed cleavages was set to 2. The result was filtered to 1% FDR at the peptide level using the Percolator algorithm integrated in Thermo Proteome Discoverer. The localization of the phosphorylation sites within the peptides was performed with the tool ptmRS, which is based on the tool phosphoRS [73].

### Structural modeling of an H2B.8-containing nucleosome

The modelled structure of an H2B.8-containing nucleosome molecule was created with SWISS-MODEL (https://swissmodel.expasy.org) using a human nucleosome as template (PDB code: 3AFA). The human H2B sequence in the nucleosome model was replaced with the *Arabidopsis* H2B.8 histone core sequence to gain insight into potential intra-nucleosomal contacts. Close-up images were obtained from the rendered model structure using PyMOL (http://www.pymol.org).

### Generation of H2B-specific antibodies

Antibodies against Arabidopsis H2Bs were raised in rabbits by immunization with following peptides coupled to KLH: H2B.1 –KTAAERPVEENKAAEKA, H2B.2 –KPAEKTPAAEPAA, H2B.3 –KPAEKAPAEEEKVAEKA, and H2B4/9/11 –KPASEKPVEEKSKAEKA. Immunization and affinity purification were done at Genescript (USA). For testing antibody specificity, nuclear extracts from 10-day-old *Arabidopsis* seedlings expressing RFP-tagged H2Bs were separated by SDS-PAGE and transferred to a 0.2 μm nitrocellulose membrane (GE Healthcare). Proteins were detected by anti-RFP (Abcam, ab62341), anti-H3 (Abcam, ab1791) or anti-H2B specific antibodies.

### Expression analysis

Total RNA was extracted using an RNeasy plus mini kit (Qiagen). Reverse transcription was performed with a RevertAid first strand synthesis kit (Thermo Scientific). Real-time quantitative PCR was carried out to measure levels of transcripts on a Roche light cycler using SYBR green PCR master mix (Roche). *UBQ10* (*AT4G05320*) or *GAPC* (*AT3G04120*) was used as an endogenous control. PCR amplification was performed using the primers listed in S2 Data. Expression profiles of H2B genes in rice, tomato and maize were extracted from Genevestigator data [49].RNA-seq data from various *Arabidopsis* tissues was analysed and described previously [19].

### ChIP-Seq

ChIP experiments using 10-day-old seedlings were performed as previously described [14]. After chromatin shearing by sonication, immunoprecipitation was carried out with anti-H3 (Abcam, ab1791) or anti-H2B specific antibodies. Sequencing libraries were prepared using a TruSeq DNA sample prep kit (Illumina), and subsequently sequenced with Illumina Hiseq 2500 to generate single-end 50 bp reads. Reads were mapped to the *Arabidopsis* genome (TAIR10) using Bowtie2 version 2.1.0 [74] and filtered for a MAPQ score > 10 using SAMtools version 1.3 [75]. Duplicate reads were removed using Picard tools MarkDuplicates version 1.141 (https://github.com/broadinstitute/picard). For data visualization, log_2_ ratio bigwig coverage files relative to input were generated using the deepTools version 2.5.0.1 utility bamCoverage with a bin size of 10bp [76]. ChIP-seq meta-profiles were generated using the EnrichedHeatmap function normalizeToMatrix [77] and plotted with a custom R script.

To analyse the enrichment of somatic H2Bs on transposon sequences, reads were re-aligned to TAIR10 using ShortStack to conserve and properly place multi-mapping reads (Johnson et al., 2016) using the—*align_only* parameter. The resulting aligned BAM files were used to generate log_2_ ratio bigwig coverage files relative to input using deepTools version 2.5.0.1 utility bamCoverage with a bin size of 10bp [76]. Transposon fragments corresponded to the coordinates of type “transposable_element” in *TAIR10_GFF3_genes_transposons*.*gff* file (www.arabidopsis.org) while Transposable Element Genes corresponded to the type “transposable_element_gene”.

## Supporting information

S1 Fig Sequences of H2Bs in different species. (A) Sequence alignment of *Arabidopsis thaliana*, (B) *Klebsormidium flaccidum* and (C) *Marchantia polymorpha* histone H2Bs. Different amino acid residues are indicated by light grey shading.
(TIF)

S2 Fig Sequences of H2Bs in different species. (A-B) Alignment of three histone H2B sequences each from *Arabidopsis thaliana*, *Marchantia polymorpha* and *Chlamydomonas reinhardtii*. Matching residues are shown as dots. Plant H2Bs show substantial sequence divergence in N-terminal tails (A) compared to the histone core domain (B). (C) Alignment of human H2B and *Arabidopsis* H2B.8 and H2B.9. The secondary structure of human H2B within the nucleosome is shown. Specific substitutions in H2B.8 are marked by blue arrows while the amino acids shown in Fig 3A are marked with red arrows.
(TIF)

S3 Fig H2B variant phylogeny in plants. (A) Amino acid alignment of select H2B.S variants from eudicots, monocots and basal angiosperms, including a non-H2B.S protein from *Arabidopsis thaliana* (H2B.6) and *Marchantia polymorpha*. H2B.S variants are indicated with light grey shading. Protein sequences that cluster at the base of the H2B.S clade (shown in B) are also included. A portion of the N-terminal domain is shown with the conserved KVVXETV motif indicated. Key substitutions in the histone core domain and extended C-terminal tail are indicated with a red asterisk (*). (B) Phylogram representation of the H2B.S clade from the maximum likelihood phylogeny shown in main Fig 1A. Major angiosperm groups are indicated with differently coloured shading, with the H2B.S variants from *Arabidopsis*, rice and maize indicated with an asterisk (*). Other highly divergent sequences from streptophyte algae and several non-angiosperm land plants are indicated with grey shading. Scale bar indicates substitutions per site. Approximate likelihood ratio test values based on a Shimodaira-Hasegawa-like procedure are indicated on nodes.
(TIF)

S4 Fig Generation and validation of H2B specific antibodies. (A) Alignment of the N-terminal of *Arabidopsis* H2B sequences. H2B.8 was excluded from the alignment. The region used to design peptide antigens for antibody production is marked by a red rectangle. (B) Details of the T-DNA insertions in the *htb* mutants used for antibody validation. Primers used for RT-PCR are indicated. (C) Relative gene expression levels in corresponding *htb* mutants. Values represent fold-changes relative to expression in wild type Col-0. Error bars represent SD from three biological replicates. *HTB3* expression was reduced but not absent in *htb3* mutant likely due to insertion of the T-DNA within its promoter region. 10-day-old seedlings were used for RNA extraction, at least 20 seedlings were used in each biological replicate. (D) Western blot detection of H2B expression using H2B specific antibodies. The protein detected in *htb4;htb9* mutant using anti-HTB4/9/11 is likely HTB11. H3 is served as loading control. (E) Western blot detection of H2B-RFP fusion proteins using H2B specific antibodies.
(TIF)

S5 Fig Controls for Fig 5. (A) Distribution of somatic H2Bs over protein-coding genes in *Arabidopsis*. Each group is colour coded using the scheme shown inlet. (B) Second and third replicate blots for the analysis shown in Fig 5F, where HA-tagged H3.1 and H3.3 were immunopurified and analysed for the presence of H2Bs.
(TIF)

S1 Data All sequences, alignments and likelihood ratio tests relating to the phylogenetic analyses performed in this study. (XLSX)

S2 Data Primers used in this study. (XLSX)
