# Supplementary figures and images for: The evolution and functional divergence of the histone H2B family in plants
Source: PLoS Genet. 2020 Jul 27;16(7):e1008964. doi: 10.1371/journal.pgen.1008964 (PMC7410336; doi:10.1371/journal.pgen.1008964)

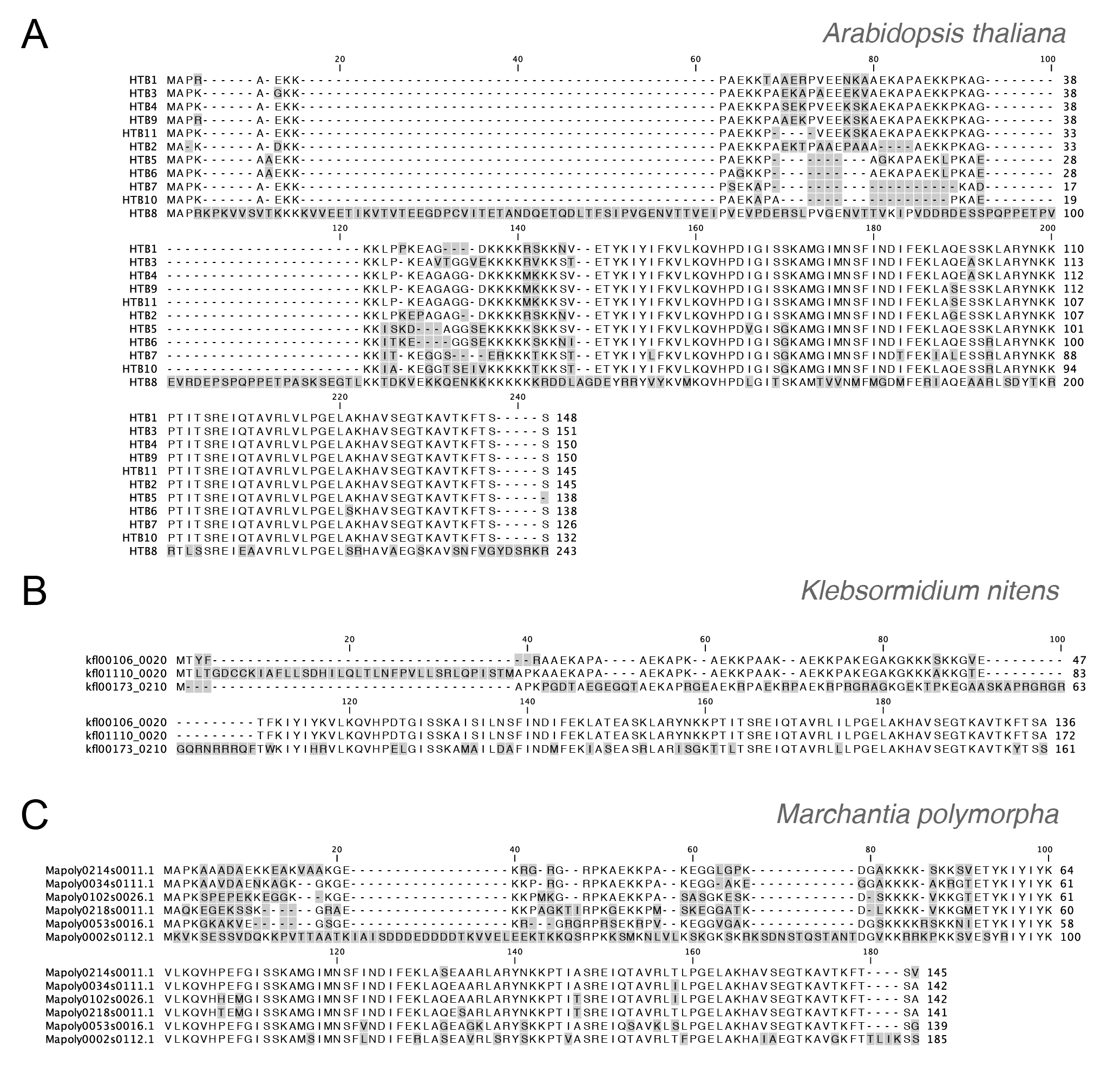

Supplement: S1 Fig — (A) Sequence alignment of Arabidopsis thaliana, (B) Klebsormidium flaccidum and (C) Marchantia polymorpha histone H2Bs. Different amino acid residues are indicated by light grey shading. (TIF) [file pgen.1008964.s001.tif]

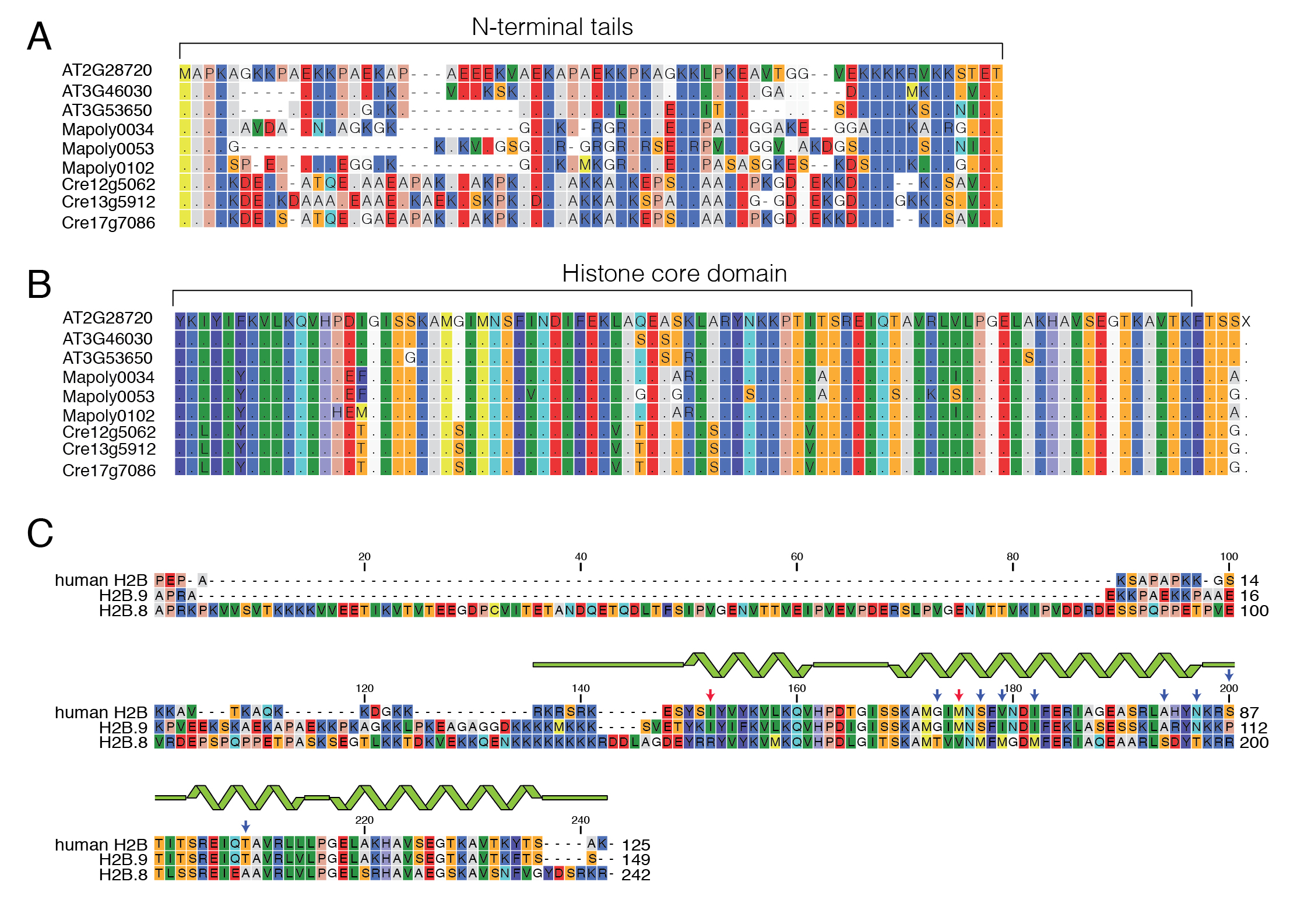

Supplement: S2 Fig — (A-B) Alignment of three histone H2B sequences each from Arabidopsis thaliana, Marchantia polymorpha and Chlamydomonas reinhardtii. Matching residues are shown as dots. Plant H2Bs show substantial sequence divergence in N-terminal tails (A) compared to the histone core domain (B). (C) Alignment of human H2B and Arabidopsis H2B.8 and H2B.9. The secondary structure of human H2B within the nucleosome is shown. Specific substitutions in H2B.8 are marked by blue arrows while the amino acids shown in Fig 3A are marked with red arrows. (TIF) [file pgen.1008964.s002.tif]

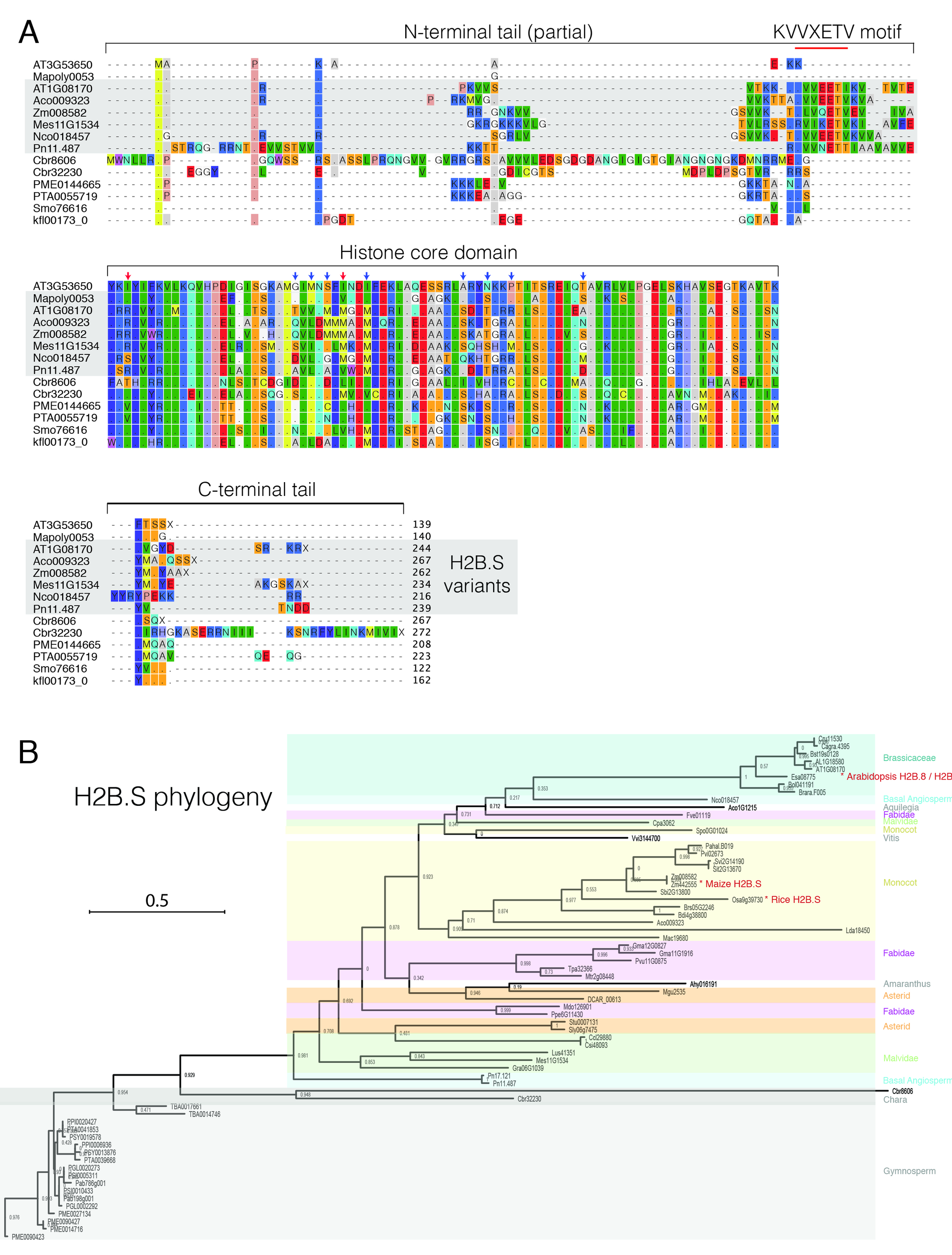

Supplement: S3 Fig — (A) Amino acid alignment of select H2B.S variants from eudicots, monocots and basal angiosperms, including a non-H2B.S protein from Arabidopsis thaliana (H2B.6) and Marchantia polymorpha. H2B.S variants are indicated with light grey shading. Protein sequences that cluster at the base of the H2B.S clade (shown in B) are also included. A portion of the N-terminal domain is shown with the conserved KVVXETV motif indicated. Key substitutions in the histone core domain and extended C-terminal tail are indicated with a red asterisk (*). (B) Phylogram representation of the H2B.S clade from the maximum likelihood phylogeny shown in main Fig 1A. Major angiosperm groups are indicated with differently coloured shading, with the H2B.S variants from Arabidopsis, rice and maize indicated with an asterisk (*). Other highly divergent sequences from streptophyte algae and several non-angiosperm land plants are indicated with grey shading. Scale bar indicates substitutions per site. Approximate likelihood ratio test values based on a Shimodaira-Hasegawa-like procedure are indicated on nodes. (TIF) [file pgen.1008964.s003.tif]

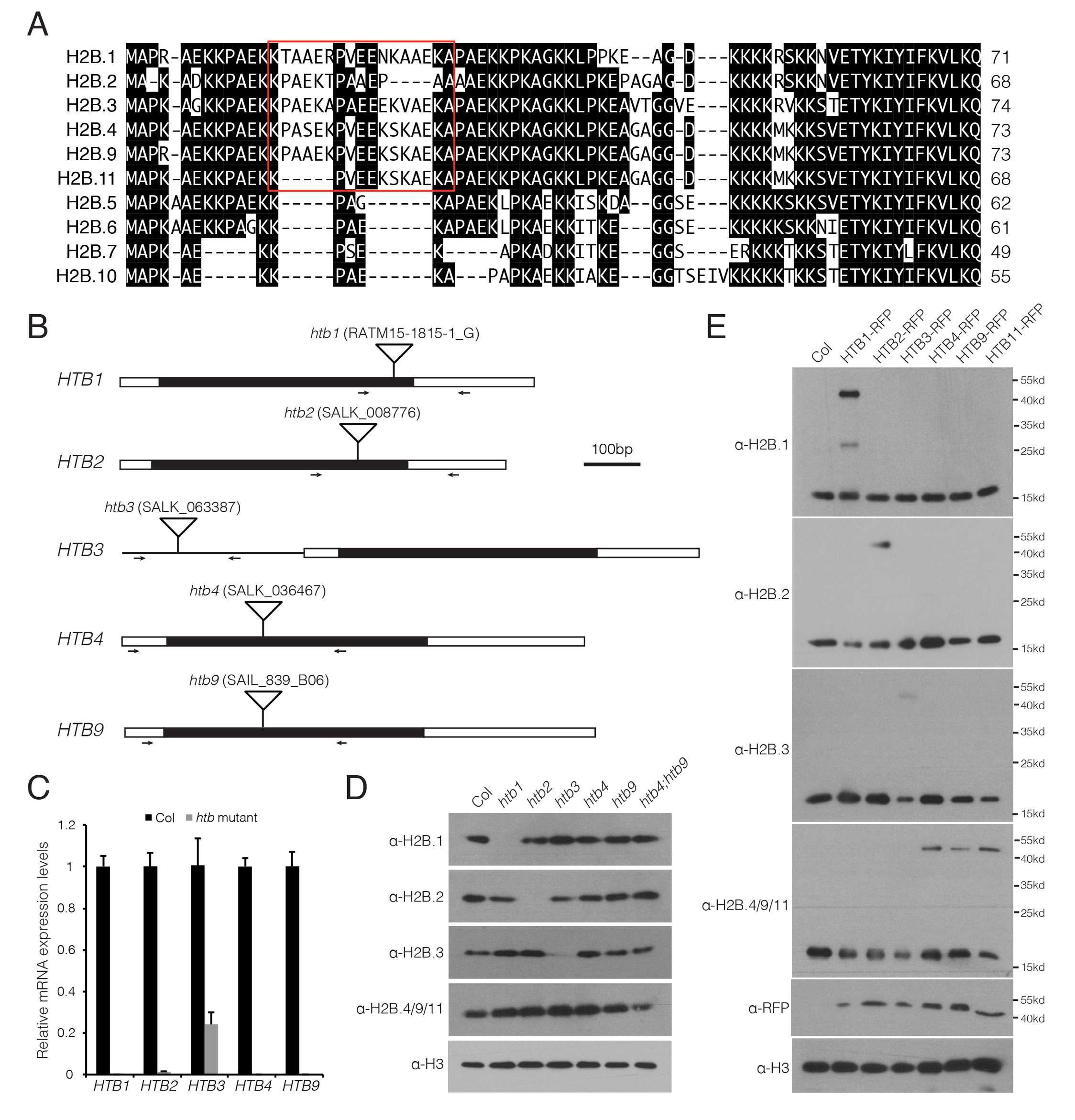

Supplement: S4 Fig — (A) Alignment of the N-terminal of Arabidopsis H2B sequences. H2B.8 was excluded from the alignment. The region used to design peptide antigens for antibody production is marked by a red rectangle. (B) Details of the T-DNA insertions in the htb mutants used for antibody validation. Primers used for RT-PCR are indicated. (C) Relative gene expression levels in corresponding htb mutants. Values represent fold-changes relative to expression in wild type Col-0. Error bars represent SD from three biological replicates. HTB3 expression was reduced but not absent in htb3 mutant likely due to insertion of the T-DNA within its promoter region. 10-day-old seedlings were used for RNA extraction, at least 20 seedlings were used in each biological replicate. (D) Western blot detection of H2B expression using H2B specific antibodies. The protein detected in htb4;htb9 mutant using anti-HTB4/9/11 is likely HTB11. H3 is served as loading control. (E) Western blot detection of H2B-RFP fusion proteins using H2B specific antibodies. (TIF) [file pgen.1008964.s004.tif]

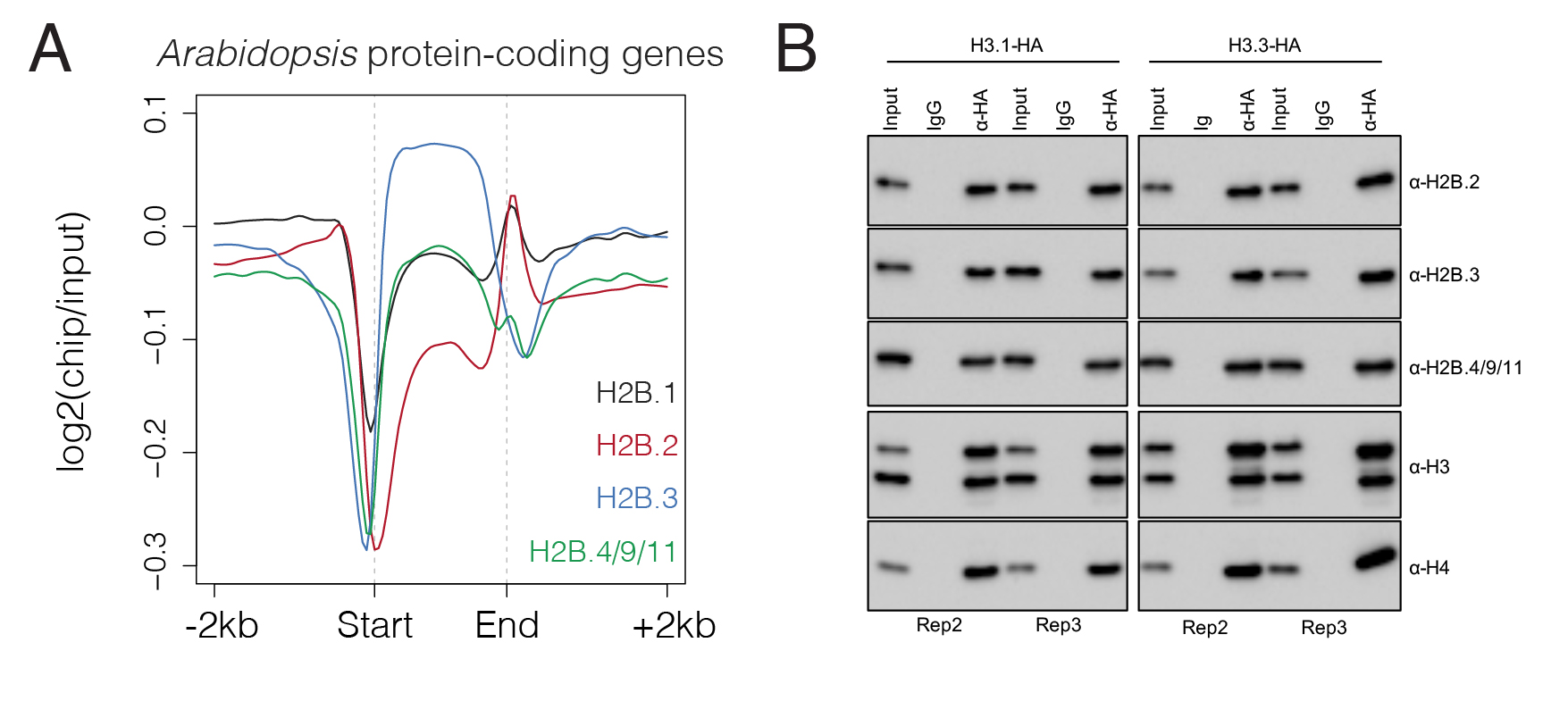

Supplement: S5 Fig — (A) Distribution of somatic H2Bs over protein-coding genes in Arabidopsis. Each group is colour coded using the scheme shown inlet. (B) Second and third replicate blots for the analysis shown in Fig 5F, where HA-tagged H3.1 and H3.3 were immunopurified and analysed for the presence of H2Bs. (TIF) [file pgen.1008964.s005.tif]
