# Supplementary material for: The evolution and functional divergence of the histone H2B family in plants
Source: PLoS Genet. 2020 Jul 27;16(7):e1008964. doi: 10.1371/journal.pgen.1008964 (PMC7410336; doi:10.1371/journal.pgen.1008964)
Supplement: Attachment — Submitted filename: 104410_1_rebuttal_1763446_q9w0y9.docx [file pgen.1008964.s008.docx]

**Reviewer #1 (Evidence, reproducibility and clarity (Required)):**

This manuscript describes a thorough study of histone H2B proteins in plants, where a significant diversification is found. They characterize various H2B isoforms by using a wide combination of strategies, including gene expression, generation of specific antibodies, microscopic analysis using RFP-tagged plants, mass spec and genome-wide ChIP. Overall, this is a quite comprehensive, though suggestive of further work in several directions. The claims are in most cases well substantiated by the results obtained (but see also below some suggestions on experimental and methodological aspects). The Introduction and the Discussion sections very valuable. This work might be greatly improved by including phenotypic descriptions of (some of) the HTB T-DNA lines identified.

**General comments**

1.The major problem refers to the Results section claiming that H2B.3 is deposited independent of DNA replication. While this is one likely possibility, this claim is too strong as it is based on circumstantial evidence. This should be assessed directly using experiments such as EdU labeling, mutants, and other strategies. Alternatively, the text in Results and Discussion, including also the title of this section, needs to be to tune down.

Response: We thank Reviewer #1 for the suggestion to use EdU labelling, which would indeed allow us to correlate cell cycle phase with expression of H2B.3. In our original manuscript, we referred to replicating cells and coupling of deposition with S-phase, terms which we now realise misled the reader by implying that H2B.3 is expressed in cycling cells but outside of the S-phase . However, our analysis points to the fact that H2B.3 expression is absent from actively dividing cells but present in differentiated non-dividing cells, suggesting cell cycle-dependent expression. We also agree with Reviewer #1 that our data is to some degree circumstantial regarding the link to S-phase deposition. We have thus toned down this section to only suggest that H2B.3 acts as a replacement histone. The title and figure legend have also been amended to reflect this.

2.It is mentioned that a genomic region containing the promoter was used for different constructs. How were such promoter regions defined? Is this based on an arbitrary size upstream of the TSS? If so, this should be indicated since those pieces of DNA may, or may not, contain the full functional promoter.

Response: We thank Reviewer #1 for pointing out this omission. To generate the H2B-RFP marker lines, we amplified a genomic DNA fragment containing the coding region and complete upstream sequence up to the nearest neighbouring gene. We have now added this important piece of information to the methods. The expression patterns of the H2B-RFP lines were all confirmed by both RNA-seq and qRT-PCR analysis.

3.The differences between "transposable elements" and "transposable fragments" should be explained in more details, as well as the consequences for the analysis discussed accordingly.

Response: We thank Reviewer #1 for pointing out a lack of clarity on these two different types of TE annotations. We have now expanded this section to explain clearly what is meant by the two classes of transposable elements, which helps to clarify the differing levels of somatic H2B enrichment we observed. Please see the specific comments relating to Figure 4 below. Additional information about the coordinates used to generate the metaplots have also been added to the methods section.

4.Description of materials used. Further details of material used, such as age, amount, tissue. Details about location of primers used will be useful in the corresponding figures, e.g. analysis of T-DNA insertions. In general, it is recommended to provided more experimental details in several places.
Response: Details of materials used in Figures 2B, 3A-C, 5A and S4C have now all be added to the figure legends. Please also see the specific comments relating to Figure 2, 3 and 5 below. The location of primers used for analyzing T-DNA insertions is now also provided in Figure S4B.

**Specific comments**

Fig. 2. How old were the seedlings used in these experiments? Amount?

Response: In Figure 2B. For seedlings, 10-day-old seedlings were used for RNA extraction, at least 20 seedlings were used in each biological replicate. For shoot apex, rosette leaf and root, tissues were dissected from 3-week-old plants, at least 30 plants were used for dissection in each biological replicate. This is now indicated in the legend

Fig. 3. Details about age, amount of material used. Also in 3B there is an extra t in the legend.

Response: In Figure 3A. Seedling, root and rosettes were collected as described in Figure 2B. For anther and pistil, at least 150 mature anthers and 30 mature pistils were used for RNA extraction. Seeds were collected based on the days after pollination, at least 500 seeds at 2DAP and 5DAP, and 250 seeds at 20DAP were used. In Figure 3B. At least 500 seeds at 5DAP and 10DAP, and 250 seeds at 15DAP and 20DAP were used in each biological replicate. In Figure3C. At least 250 seeds at each time point were used in each biological replicate.

The extra “t” has been removed from the legend.

Fig. 4. Discuss the significance of changes observed in the chromatin pattern of H2B.3 between transposable elements and transposable fragments.

Response: We thank Reviewer #1 for this suggestion. As discussed in point 4 above, we have now added important points about what transposable element genes are and how they are similar to protein-coding genes, in the sense that they often have a typical gene structure with a flanking promoter and long gene body, and thus have the potential to undergo transcription. In light of this, we can show how H2B.3 has a pattern over TE genes similar to that of protein-coding genes, particularly non-transcribed genes, which is mirrored by a similarly matching pattern for H2B.1 and H2B.2. This has helped to strengthen our observations, where show how H2B.3 deposition appears to correlate with transcription, while H2B.1 and H2B.2 have a preference for transcriptionally inactive regions of the genome.

Panel 4D, "It shows somatic H2Bs and histone H3 over genes sorted by quantiles of expression level in Arabidopsis seedlings". This sentence seems a bit obscure. Please, reword, perhaps providing examples to clarify the conclusions.

Response: This has now been changed to “somatic H2Bs and histone H3 over genes grouped by their level of expression in *Arabidopsis* seedlings.”

Fig. 5. Panel 5A. What is the reason/advantage of normalizing to the values in the shoot apex? Please, justify or modify the normalization criteria. There seems to be significant differences between data in 5A (similar in SAM and leaves) and in 2A-B (higher in leaves) in the expression levels for HTB3. What is the reason for this discrepancy and discuss.

Response: We thank Reviewer #1 for this question. For Figure 5A, gene expression levels were normalized to that in the shoot apex so as to better visualize and compare the different H2B genes. Other normalization methods, such as the normalization to UBQ10 used in Figure 2B, will generate highly variable values due to the very different expression levels of each H2B gene. Moreover, the two tissues arise from 3-week-old plants grown on ½ MS plates (Figure 2B) and 4-week-old plants grown on soil (Figure 5A) - these different developmental stages and growth conditions may cause further variations. To circumvent this, we normalise H2B mRNA levels in young and mature leaf relative to the shoot apex – this ensures we can visualise differences and directly compare expression among the different H2Bs more clearly. Our results are further validated by *HTB3* expression in suspension cultures, where it again has the highest expression outside of S-phase.

Panel 5E. The differences claimed as significant need to be justify quantitatively better than shown in 5F.

Response: As requested by Reviewer #1, we have now replicated this analysis a total of three times to better quantify the differences in H3.1 and H3.3 association at the nucleosome level. Panel 5F has been updated to reflect this to include the range of standard error while the other gel images have been added to Figure S5B.

*Reviewer #1 (Significance (Required)):*

The study constitutes a significant conceptual advance in the field towards a better understanding of the function and diversification of a key family of histone proteins. In this context, the study is useful for a wide audience.

Response: We would like thank Reviewer #1 for the useful suggestions that have improved our manuscript, which they kindly recognise as valuable and of broad interest to a wide audience.

**Reviewer #2 (Evidence, reproducibility and clarity (Required)):**

**Summary:**

In this manuscript, Jiang et al. report an analysis of H2B histones in Arabidopsis using evolutionary analyses, tissue-specific expression and in-vivo chromatin profiling. Their main finding is the tissue restriction of H2B variants during development, and the differential localization of somatic H2Bs. They also describe a seed specific H2B.8 variants, found in angiosperms, and the replication independent incorporation of H2B.3. There is no pre-print available for this manuscript.

**Major comments:**

*Are the key conclusions convincing?*

The identification of H2B variants using phylogeny is convincing, with some minor concerns detailed below. The restriction of H2B.8 to angiosperm and its "rapid evolution" seem more preliminary. The tissue expression is convincing. The chromatin profiling is convincing, with some questions below. The incorporation of H2B.3 outside of the cell cycle, while indirect, is also convincing.


*Should the authors qualify some of their claims as preliminary or speculative, or remove them altogether?*

1) The claims about the common origin and rapid evolution of all H2B.8 in angiosperm, although attractive, is not necessarily well supported by the protein phylogenies presented. The authors seem careful about this interpretation in the main text, but should make sure to speculate in the abstract. H2B.8 seems to have a novel domain and be divergent from other H2Bs. Their work clearly shows that. In addition, there are still unanswered questions regarding the relative placement of each H2B group described, and this blurs interpretations of the origins of these proteins. The authors should make sure that it remains speculative.

Response: We agree with Reviewer #2 that the origins of each H2B group should remain speculative and were careful to balance this claim in the main text, as they rightly point out. We have further adjusted the text in the abstract to ensure this is the case there also.

2) "Our study has highlighted the high sequence divergence of H2B proteins in plants compared with most animals." Can the authors explain how this stems as a result of their analysis considering that there is no analysis of animals H2Bs (but for Human).

Response: We agree with Reviewer #2 that this was overstated as we did not perform an extensive analysis of animal H2Bs. Our work has rather highlighted the significant expansion of H2Bs in plants, which is largely driven by subtle but pervasive sequence variation in their N-terminal tails. We have thus reworked this sentence and adjusted other statements in the manuscript to refocus the impact of our work – which addresses the evolutionary diversification and expansion of H2B proteins in the plant kingdom.

3) While the claims about heterochromatin and actively transcribed chromatin seems well supported, the specific genome localization of the variants (transposons vs. other repeats vs. genes) is more preliminary - for example it is not discussed whether the age and expression of repeats affects the localization of the histones.

Response: We thank Reviewer #2 for this comment. The purpose of our analysis was to determine any obvious differences in the genomic localisation of the somatic H2Bs, which are clear and well supported as they kindly point out. We have also been careful to validate these observations through independent biochemical experiments at the mono-nucleosome level (Fig. 4E-F and Fig. 5E-F), which largely substantiate our claims. We address their point about TE age and repeat transcription below.

*Would additional experiments be essential to support the claims of the paper? Request additional experiments only where necessary to evaluate the paper as it is, and do not ask authors to open new lines of experimentation.*

1) Can the authors address the later point abut repeat transcription and the localization of the
variants as they did for genes.

Response: For our ChIP-seq analysis, we only considered non-duplicate reads with a mapQ score greater than 10. This is a conservative approach to ensure that only uniquely mapping reads are considered and to also ensure no PCR duplicates conflate the analysis. Mapping the reads to repeats would require specialised algorithms and, while indeed interesting, goes beyond our goal of determining bulk differences in genomic H2B deposition. We should also point out that *Arabidopsis* has a very small and compact genome with a limited repeat space. A definitive study of TE age is also still not available, thus making it infeasible to consider these two interesting suggestions.

2) Can the author address if the RFP fusions are found in nucleosomes and show the sequence validation of their constructs in planta? It is not clear from the methods what extraction was used to probe for their chromatin association.

Response: The H2B-RFP marker lines were used solely to assess the expression pattern of the H2Bs *in planta*, which we importantly also assessed and verified using both RNA-seq and qRT-PCR analysis. They were not used in any of the biochemical analyses (i.e. for ChIP-seq, nucleosome IPs), for which we instead used antibodies specific for each somatic H2B. In the case of chromatin extraction, we used micrococcal nuclease digestion to generate mono-nucleosome to assess chromatin association of the different H2Bs at the nucleosome level. For ChIP-seq, we used formaldehyde crosslinking and sonication to generate soluble chromatin fragments for chromatin IP. These details have all been added to the methods.

*Are the suggested experiments realistic for the authors? It would help if you could add an estimated cost and time investment for substantial experiments.*

Yes. These are only clarification points on existing data.

*Are the data and the methods presented in such a way that they can be reproduced?
Yes, with the following questions:*

1) What is the source of RNA-seq in Arabidopsis used in Figure 2? I can't seem to find this information.

Response: We thank Reviewer #2 for spotting this accidental omission. The Arabidopsis RNA-seq data used in Figure 2 was compiled and analysed in our recent publication (Borg et al., 2020). The information and relevant reference have now been added to the methods section.

2) Can the author provide more details about repeat mapping and annotation? This information is missing from the methods.

Response: This important information has now been added to the ChIP-seq analysis section of the methods.

*Are the experiments adequately replicated and statistical analysis adequate?*

Yes, with the following questions:

1) Can the authors comments on long-branch attraction in their neighbor joining trees in Figure 1?

Response: We thank reviewer #2 for noting this. The subgroup of H2B.7/10 identified in Figure 1A is likely the result of long-branch attraction from H2B.10, a result that is not recapitulated in the following maximum likelihood phylogenies shown in Figure 1B-D. As maximum likelihood phylogenies are less susceptible to long-branch attraction, the latter result is likely more reflective of the true relationships between these proteins. We have thus removed Figure 1A and instead note the subgroups of *Arabidopsis* H2Bs as is seen in the maximum likelihood phylogenies. While there may be additional instances of long-branch attraction in these phylogenies, notably with one *Lilium* H2B, we have retained these sequences for completeness as the overall tree structure does not vary without them.

**Minor comments:**

Specific experimental issues that are easily addressable

1) When making large phylogenies, can the author use yeast or human H2B to artificially root their trees? It would provide some context with respect to Talbert 2012.

Response: We have included a small number of Chlorophyte H2B sequences that may be used as an outgroup to Streptophyte H2Bs (land plants plus non-Chlorophyte green algae). These sequences are all found within the “non-angiosperm” clade in Figure 1A (formerly Figure 1B). We opted to show an unrooted tree as it better highlights the clades of interest.

2) On this note, can the authors address which of the histones described is canonical H2B in Arabidopsis (H2B.1?)?

Response: We thank Reviewer #2 for raising this important point. Our phylogenetic analysis consistently groups H2Bs by each major group in the plant kingdom. No consistent pattern emerges that would make it easy for us to identify a group of ‘canonical’ H2Bs. This term is often used for a group of H2As that are not assignable as a distinct variant like H2A.X, H2A.Z or H2A.W in plants. We believe that this term to be rather ambiguous. Both our work here on H2Bs and our other ongoing work with H2A variants argues that ‘canonical’ histones do not really exist and that it is an anecdotal term used to classify ‘non-specialised’ histones with no obvious assigned features. We are thus reluctant to assign one or more of the H2Bs as canonical.

3) "Male germline-specific H2B isoforms were previously described in lily, a monocot species (Ueda et al., 2000), indicating that tissue-specific H2Bs might be common among flowering plants but have likely arisen independently during evolution, constituting a putative new class of H2B variants". How does it compare to H2B.8 or the rest of the H2Bs found in Arabidopsis?

Response: We thank Reviewer #2 for raising the point about lily H2Bs. Four full-length H2B sequences were found in a publication that we now cite and that have been incorporated into the analysis in Figure 1A. Of the two male germline specific H2Bs, one grouped with other monocot H2B.S sequences, while the second grouped with eudicot H2Bs, albeit on an extremely long branch. The latter lily sequence was highly divergent from all other plant H2Bs, which makes it difficult to assess its relationship with the other *Arabidopsis* H2Bs. The other two lily H2Bs formed part of the "vascular plant" clade, apart from all other monocot H2Bs.

*Are prior studies referenced appropriately?*

Seems appropriate from my expertise.

*Are the text and figures clear and accurate?*

Yes, with the suggestions in the next section.

*Do you have suggestions that would help the authors improve the presentation of their data and conclusions?*

1) Can the authors refer to histone variants as opposed to isoforms. It would help the reader understand that these are separate genes with their own regulation and evolutionary histories. Unfortunately "isoform" can be mis-leading, e.g. splice variants leading to the production of protein isoforms. I found myself confused by the term isoform in the tittle and abstract.

Response: We thank Reviewer #2 for raising this. Upon considering this point and re-reading the manuscript, we agree that the term isoform might create confusion. We have thus removed the term “isoform” from our revised manuscript, and only refer to the H2B.S class as *bona fide* variants given their distinct expression pattern and evolutionary history.

2) Is there a need to rename H2B.8: H2B.S (in the abstract). It might confuse readers that are well versed in plant histones (cf. Talbert 2012)

Response: In our manuscript, we are only classifying H2B.8 to be part of the H2B.S class of histone variants - we do not plan or suggest renaming H2B.8 itself. In the abstract, we also do not refer to H2B.8 but rather to our discovery and reasoning for a new class of histone variants that associate with compact chromatin of dry seeds in multiple flowering plant species. We hope that this clarifies this issue.

3) Figure 1B,C and D are hard to interpret. What is the coloring? What are the group being shown. Maybe switch to a rooted view to orient the reader, or split into 2 figures.

Response: We agree with Reviewer #2 that we failed to clarify properly what this coloring meant. With the exception of H2B.S, we coloured the different groups in Figure 1 based on angiosperm group-specific clades and family-specific clades. This clarification has now been added to the Figure legend. We are reluctant about using a rooted phylogeny because this will simply root the tree somewhere inside the non-angiosperm clade – a non-rooted tree is thus more impartial and highlights better how the separations are driven by group-specific divergence.

4) "Eight out of the ten amino acids in human H2B were conserved in H2B.9 but not in H2B.8 (Figure S2C) and all faced histone H2A, H2B.8 itself or H4 (Figure 3H)." Can the author clarify what this means?

Response: We thank Reviewer #2 for suggesting more clarification on this point. This sentence has now been broken down further as follows: “Eight out of the ten amino acids in human H2B were conserved in H2B.9 but not in H2B.8 (Figure S2C). These H2B.8-specific amino acids were all orientated towards the inner core of the nucleosome and faced histone H2A, H4 or H2B.8 itself (Figure 3H).”

*Reviewer #2 (Significance (Required)):*

*Describe the nature and significance of the advance (e.g. conceptual, technical, clinical) for the field.*

This study provides conceptual advances. This study is an improvement on the characterization of histone variant in plants.

*State what audience might be interested in and influenced by the reported findings.*

Researchers interested in histones and chromatin regulation during plant development.

*Define your field of expertise with a few keywords to help the authors contextualize your point of view. Indicate if there are any parts of the paper that you do not have sufficient expertise to evaluate.*

Key words: histone variants.

Part of the paper with no sufficient expertise: protein biochemistry and plant development.

Response: We would like to thank Reviewer #2 for raising key points and clarifications that have improved our revised manuscript and for also recognising the value of our work to researchers interested in histones and chromatin regulation.

**Reviewer #3 (Evidence, reproducibility and clarity (Required)):**

Histone variants have critical roles in chromosome structure and function. While many variants in H2A and H3 have been identified and characterized in eukaryotes, the array of known variants in H2B and H4 is limited. Using phylogenetic analyses, Jiang et al data support previous results showing H2B diversification in plants. Through genomics and biochemical approaches, they perform initial characterization of some of these H2B variants and show tissue-specific expression, and even different patterns of localization in chromatin. The experiments and analyses are generally appropriately interpreted.

**Major comments:**

-The analyses and experiments are generally convincing.

-There is already a substantial amount of work in this report, no further experiments are essential.

-Data appear to be reproducible and when possible replicates were performed

-Statements are generally conservative, though rapid evolution would be better supported by dN/dS analyses (e.g. Molaro AM et al Genome Res 2018).

Response: We are currently testing if H2B.S clade has relaxed purifying selection using a dN/dS analysis and will provide the result at the final stages of revisions.

-Introduction should be shortened, main point is that few H2B variants have been identified in eukaryotes, in contrast to H2A and H4

Response: As suggested, we have substantially shortened the introduction in our revised manuscript.

**Minor comments:**

-Data indicating replication-independent deposition of H2B.3 are interesting, but this given the relatively high degree of similarity between H2B.3 and other H2Bs, is this just related to expression outside of S-phase? Does ectopic expression of H2B.3 in rapidly dividing cells lead to incorporation in a replication-coupled manner, and does expression of H2B.2 or 4/9/11 in post-mitotic cells result in deposition in chromatin? H3.3 has specific chaperones and those are likely driving the observed deposition pattern, there may be little functional consequence to H2B.3 deposition as opposed to H2B.2 or 4/9/11. These experiments are not essential for the paper.

Response: We thank Reviewer #3 for raising these interesting questions and suggestions for future work into the role of H2B.3, which they also agree are beyond the scope of this paper. Indeed, as we state in the discussion, H2B.3 shows little sequence divergence compared to the other H2Bs. It is thus likely that these differences have arisen from distinct upstream regulatory control but which also point to novel deposition mechanisms. Future experiments will help to test these interesting hypotheses.

-References are appropriate, Draizen EJ et al Database 2016 should be cited for HistoneDB 2.0 since readers can quickly view some of the relevant H2B sequences and alignments there.

Response: We have now added this important reference to the very beginning of the results section.

-Text is clear and well-written. Figures are clear, some of the colored alignments obscure the amino acid letters.

*Reviewer #3 (Significance (Required)):*

This study more comprehensively characterizes the already reported diversification of H2B in plants. While the phylogenetic analyses themselves are interesting, the demonstration of a subset of the variants having germ cell-specific expression and evidence suggesting a role in chromatin compaction are intriguing. Further, the data suggesting H2B.3 is deposited in a replication-independent manner are exciting, but whether there is a functional consequence to H2B.3 deposition as opposed to other H2B variants is not known but beyond the scope of this paper. Though the plant-specific focus will limit readership, this would be of interest to evolutionary biologists and chromatin biologists.

Response: We would like to thank Reviewer #3 for recognising the depth of the experiments and analyses we have performed and for noting the broad interest this work will have to evolutionary and chromatin biologists beyond the plant field.
