# Supplementary material for: The evolution and functional divergence of the histone H2B family in plants
Source: PLoS Genet. 2020 Jul 27;16(7):e1008964. doi: 10.1371/journal.pgen.1008964 (PMC7410336; doi:10.1371/journal.pgen.1008964)
Supplement: Attachment — Submitted filename: Reviewer_comments_2.docx [file pgen.1008964.s009.docx]

(1) Please add the dN/dS analysis as requested by Reviewer 3. This could be useful to see if there are any branch-specific changes in selective constraint. Optional: If so, statistical significance for changes in selective constraint his could be evaluated under PAML or HyPhY programs.

We have completed the dN/dS analysis as requested and added the results and statistical tests to Supplemental Data 1. We indeed found a statistically significant relaxation of purifying selection for the H2B.S clade, but not Eudicot groups 1 and 2. However, we found no significant relaxation of selection on specific sites in the H2B.S lineage, as indicated by the branch-sites test. We would like to thank Reviewer 3 for their suggestion.

(2) We understand that your RNA-seq and ChiP-seq analyses only focused on uniuely mapping reads. However, since the repeat classes of Arabidopsis are relatively well-known, it should be possible to map multiply mapping reads to specific repeat classes using a repeat library approach, as suggested by Reviewer 2. This would allow you to make a stronger statement about whether there are specific repeats that are affected by the histone variants.

We used previously published RNA-seq data (Borg et al., 2020 – Nature Cell Biology) solely to assess the expression of the H2B variants among different *Arabidopsis* tissues. This allowed us to assess their developmental profiles and pinpoint the reproductive class of H2Bs. As requested, we have re-aligned the H2B ChIP-seq data using ShortStack, a dedicated alignment algorithm that used local genomic context to guide decisions on proper placements of multi-mapping reads. We have used these new alignments to generate new plots of H2B enrichment over repeats i.e. transposon fragments and transposable element genes.

Although subtle, the ShortStack alignment has accentuated the enrichment between the somatic H2Bs (see figure below) compared with our original Bowtie2 alignment that discarded multi-mapping reads. This new analysis has confirmed H2B.2’s preference for silenced repeat sequences, further strengthening our initial conclusions. These plots have now been updated in the revised manuscript. We would thus like to thank reviewer #2 for this insightful suggestion.

(3) OPTIONAL: rooting with a non-plant H2B could allow you to place the location of the outgroup via an arrow on the unrooted tree. This way you can keep the unrooted tree format (which we prefer also) but indicatethe relative ancestry of the histone variants, which is what Reviewer 3 was trying to suggest.

We have constructed phylogenies with human H2B to place the location of the outgroup, but have found its placement to be uninformative. In the Plant H2B phylogeny, human H2B is within a clade of gymnosperm H2Bs, while in the Eudicot phylogeny it is nested within a clade of Asterid H2Bs. In both cases, we believe this placement to be erroneous, most likely due to long branch attraction. Therefore, we have opted to not include human H2B in the final phylogenies, though we have added an arrow in Figure 1A to indicate the Chlorophyte algae H2Bs, and thus the branching leading to all Streptophyte algae and land plant H2Bs.
